# Antibiofilm properties of bioactive compounds from *Actinomycetes* against foodborne and fish pathogens

**DOI:** 10.1038/s41598-022-23455-8

**Published:** 2022-11-03

**Authors:** Tracy Miller, Diana Elizabeth Waturangi, Yogiara

**Affiliations:** Biotechnology Department, Faculty of Biotechnology, Atma Jaya Catholic University of Indonesia, Jenderal Sudirman 51 Street, South Jakarta, DKI Jakarta 12930 Indonesia

**Keywords:** Biotechnology, Microbiology

## Abstract

In nature, bacteria can form biofilms, multi-layered structures that adhere microbial populations to solid surfaces by exopolysaccharides, proteins, and nucleic acids. In addition to causing foodborne infections, biofilms can be a major problem in aquaculture. *Actinomycetes* extracts have previously demonstrated antibiofilm activity against multiple foodborne and fish pathogens, and further characterization of these extracts is needed. In this study, we identified the chemical structures and antibiofilm properties of four extracts and determined the genetic similarity of the isolates to known *Streptomyces* isolates. We found that several extracts contained multiple antibiofilm compounds, and the antibiofilm activities of all extracts were most stable at pH 6. Furthermore, the antibiofilm inhibition and destruction activities of the isolates were stable at different temperatures. All of crude extracts demonstrated activity against biofilms formed by foodborne and fish pathogens on the surface of stainless-steel coupons as well as polystyrene that commonly used in industrial equipment. Using PCR 16S-rRNA gene and DNA sequencing analysis, the four *Actinomycetes* isolates were found to be 99% (1 AC), 97% (20 PM), 95% (16 PM), and 85% (18 PM) similar to *Streptomyces*. Biofilm structure were analyzed using Scanning Electron Microscopy coupled with Energy-Dispersive Spectrometry analysis. Coniine/(S)-2-propylpiperidine was the most active fraction of the crude extracts of the 1 AC, 20 PM, and 16 PM isolates, and piperidine, 2-(tetrahydro-2-furanyl) was most active in the 18 PM isolate.

## Introduction

Foodborne bacteria present a wide range of public health concerns around the world. Foodborne diseases have become a serious problem in a variety of food industries and are often associated with microbial infections of workers and consumers. Biofilm is thought to be responsible for 80 percent of all bacterial infections^1^. They also pose a serious threat to marine aquaculture^2^.

Antibiotics are ineffective in treating biofilm-associated infections due to the rapid development of bacterial resistance to all antibiotic classes. Other factors contributing to resistance include the nature and structure of the biofilm, nutrient and oxygen availability for bacterial cells, and intrinsic and acquired bacterial resistance^3,4^.

*Actinomycete* bacteria are known for their production of bioactive compounds, a quality widely exploited in the pharmaceutical, agricultural, and food industries^5–8^. Members of this bacterial group have demonstrated the ability to produce active antibiofilm agents^9–11^. Two examples of this are *Streptomyces albus* and *S. akiyosheinsis*, respectively, inhibiting the biofilms of *Vibrio harveyi* and *Staphylococcus aureus*^12^.

Compounds isolated from *Actinomycete* bacteria may provide alternatives to effectively control biofilms, as they may be much less likely to promote resistance than conventional antibiotics. The aim of this research was to genetically characterize *Actinomycete* isolates and identify specific properties of their antibiofilm compounds that inhibit and destroy biofilms formed by foodborne and fish pathogens.

## Results

### Bioactive compound characterization

**Figure 1:**
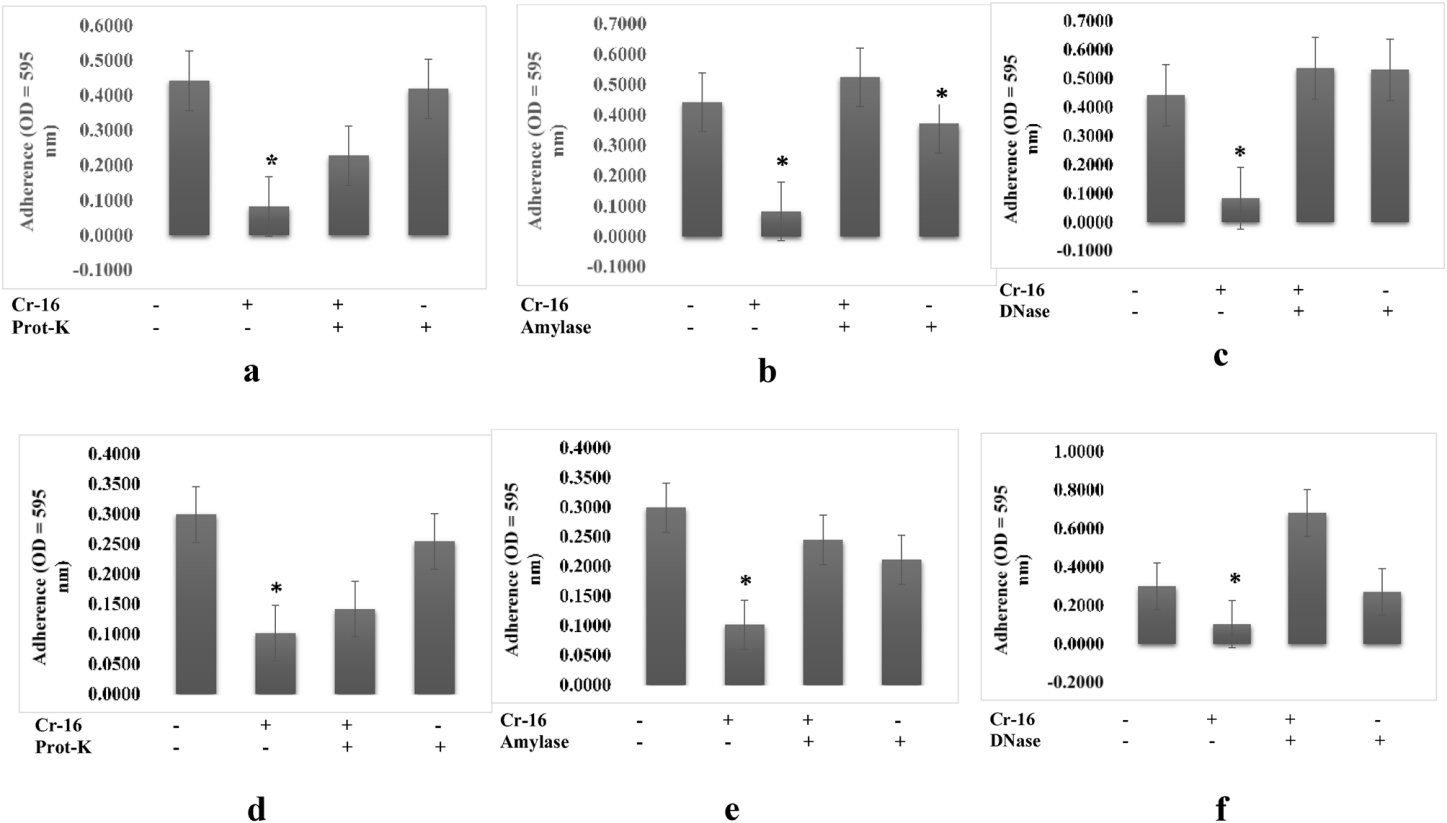
Characterization of extracts produced by 16PM isolate (**a**–**c**) which were responsible for the antibiofilm activity towards biofilm structure of *A. hydrophila* and (**d**–**f**) which were responsible for the antibiofilm activity towards biofilm structure of *V. harveyi* by treating the extract with amylase (1 mg/mL), proteinase K (1 mg/mL), and DNase I (100 mg/mL). (n = 6 replications; vertical bars are standard errors; *: significantly different at p < 0.05).

Pre-treatment of the 16 PM extract with proteinase-K, amylase, and DNAse decreased its antibiofilm activity against biofilm of *A. hydrophila* (Fig. 1a–c) and *V. harveyi* (Fig. 1d–f). These results indicate that the 16 PM extract contained three components (polysaccharides, protein, and nucleic acid) as major component for their antibiofilm properties which were effective against biofilm of *A. hydrophila* and *V. harveyi*.

**Figure 2:**
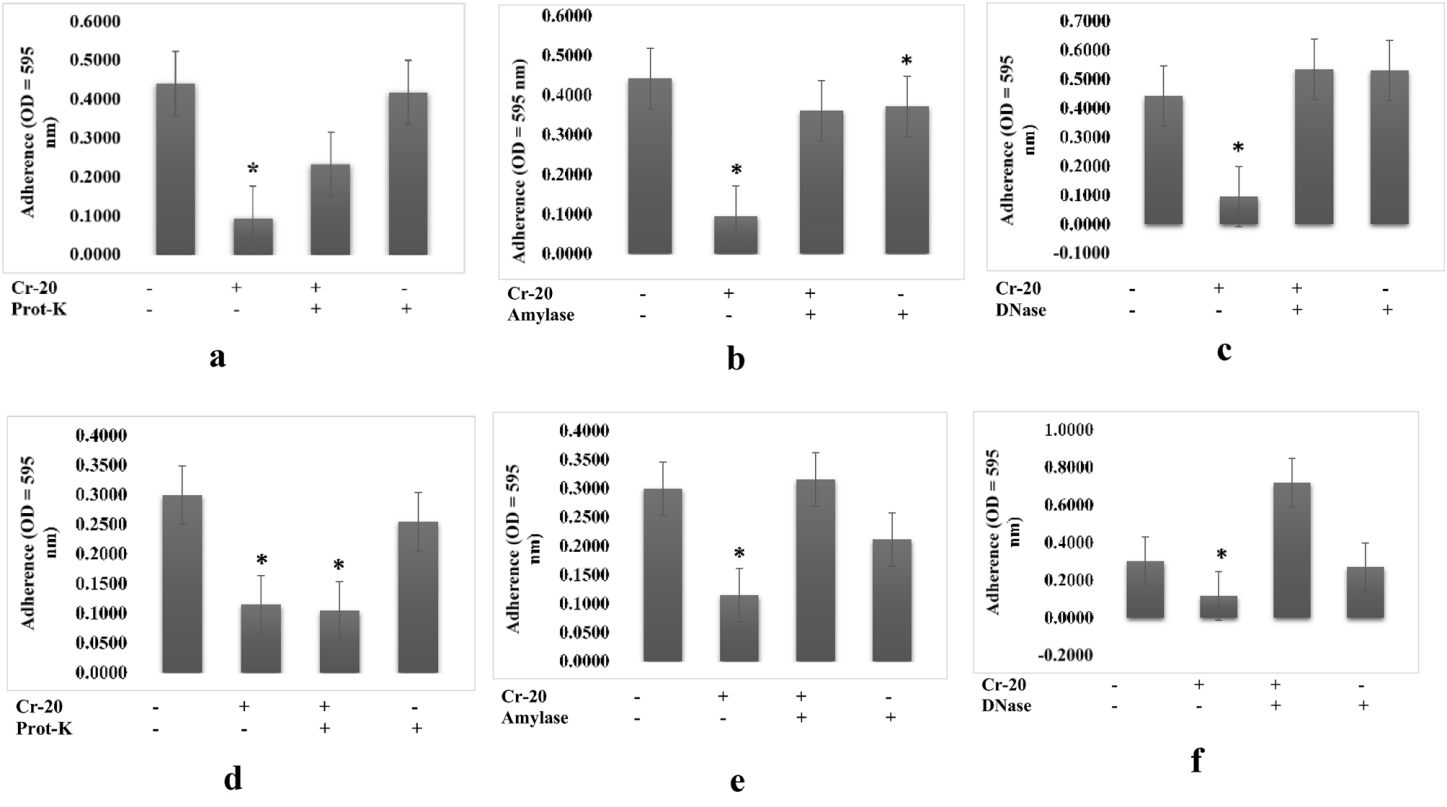
Characterization of extracts produced by 20PM isolate (**a**–**c**) which were responsible for the antibiofilm activity towards biofilm structure of *A. hydrophila* and (**d**–**f**) which were responsible for the antibiofilm activity towards biofilm structure of *V. harveyi* by treating the extract with amylase (1 mg/mL), proteinase K (1 mg/mL), and DNase I (100 mg/mL). (n = 6 replications; vertical bars are standard errors; *: significantly different at p < 0.05).

Pre-treatment of the 20 PM extract with proteinase-K, DNAse, and amylase decreased its antibiofilm activity against biofilm of *A. hydrophila* (Fig. 2a–c), while only DNAse and amylase decreased its antibiofilm activity against biofilm of *V. harveyi* (Fig. 2e, f). Pre-treatment with proteinase-K showed no effect on its antibiofilm activity (Fig. 2d).

**Figure 3:**
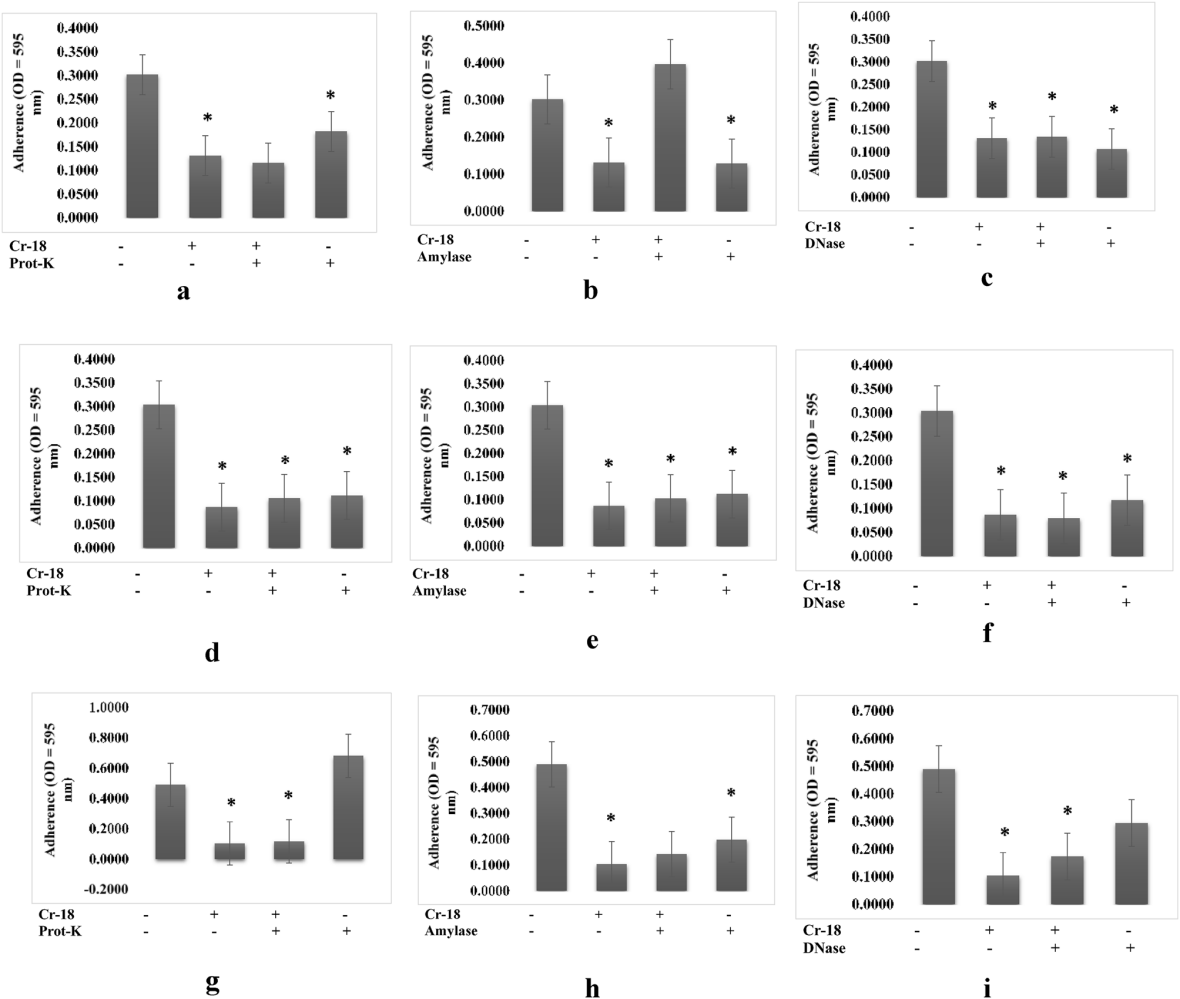
Characterization of extracts produced by 18PM isolate (**a**–**c**) which were responsible for the antibiofilm activity towards biofilm structure of *S. putrefaciens,* (**d**–**f**) which were responsible for the antibiofilm activity towards biofilm structure of *B. cereus,* (**g**–**i**) which were responsible for the antibiofilm activity towards biofilm structure of *B. subtilis,* by treating the extract with, proteinase K (1 mg/mL), amylase (1 mg/mL), and DNase I (100 mg/mL). (n = 6 replications; vertical bars are standard errors; *: significantly different at p < 0.05).

Pre-treatment of the 18 PM extract with amylase decreased its antibiofilm activity against biofilm of *Shewanella putrefaciens* (Fig. 3b), whereas pre-treatment with proteinase-K and DNase did not affect biofilm formation (Fig. 3a, c), indicating that polysaccharides were responsible for the antibiofilm activity. Pre-treatment with proteinase-K and amylase decreased the antibiofilm activity of 18 PM extracts against the *Bacillus cereus* biofilms (Fig. 3d, e), but DNase pre-treatment showed no effect (Fig. 3f). Moreover, pre-treatment with amylase and DNase decreased the antibiofilm activity of 18 PM extracts against biofilm of *B*. *subtilis* (Fig. 3h, i), but pre-treatment with proteinase-K showed no effect on those activities (Fig. 3g).

**Figure 4:**
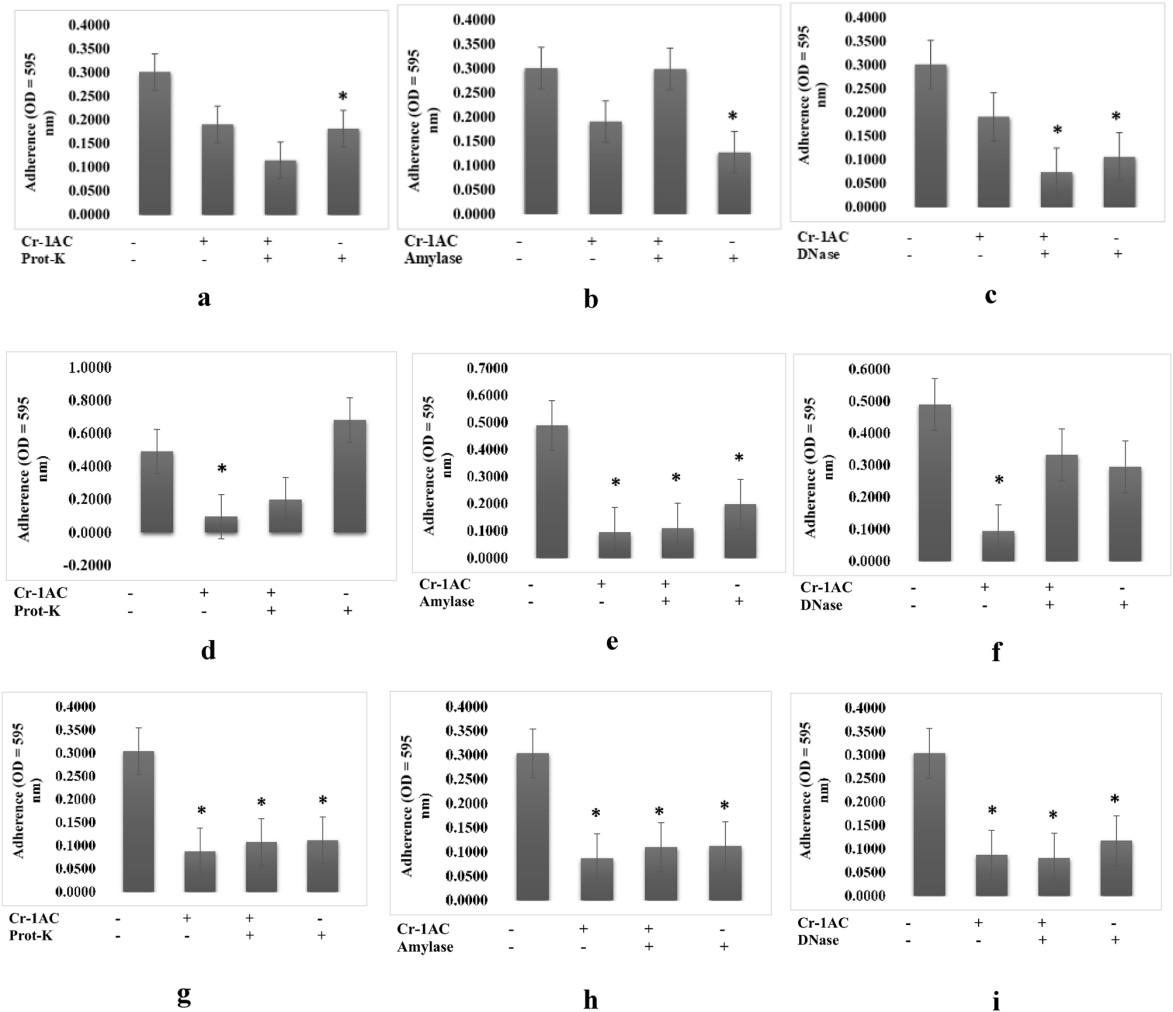
Characterization of extracts produced by 1AC isolate (**a**–**c**) which were responsible for the antibiofilm activity towards biofilm structure of *S. putrefaciens,* (**d**–**f**) which were responsible for the antibiofilm activity towards biofilm structure of *B. cereus,* (**g**–**i**) which were responsible for the antibiofilm activity towards biofilm structure of *B. subtilis,* by treating the extract with amylase (1 mg/mL), proteinase K (1 mg/mL), and DNase I (100 mg/mL). (n = 6 replications; vertical bars are standard errors; *: significantly different at p < 0.05).

Pre-treatment of the 1 AC extract with amylase decreased its antibiofilm activity against biofilm of *S. putrefaciens* (Fig. 4b), whereas pre-treatment of proteinase-K and DNase had no affect (Fig. 4a, c). Pre-treatment of the 1 AC extract with proteinase-K and amylase decreased its antibiofilm activity against biofilm of *B. cereus* (Fig. 4d, e), while pre-treatment with DNase showed no effect (Fig. 4f). Finally, while pre-treatment of the 1 AC extract with proteinase-K and DNase decreased its antibiofilm activity against biofilm of *B. subtilis* (Fig. 4g, i), pre-treatment with amylase showed no effect (Fig. 4h).

### pH Stability

**Figure 5:**
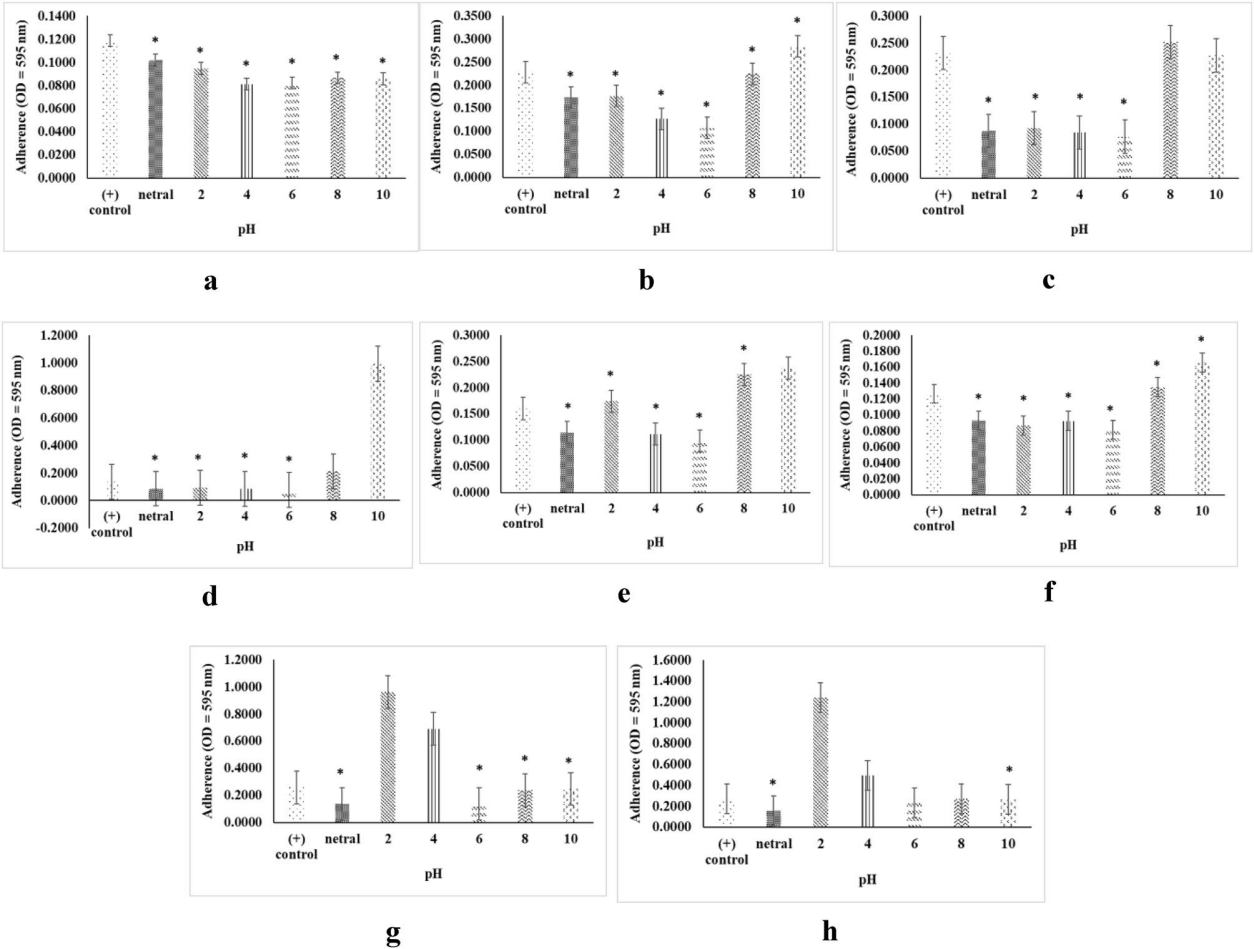
Stability of inhibition activity (**a**) 16PM, (**b**) 20PM, (**c**) 18PM, (**d**) 1AC and destruction activity (e) 16PM, (**f**) 20PM, (**g**) 18PM, and (**h**) 1AC (n = 6 replications; vertical bars are standard errors; *: significantly different at p < 0.05).

Within the range of pH 2–10, all of crude extracts demonstrated the most stable biofilm inhibition and destruction activities at pH 6 (Fig. 5).

### Thermal stability

**Figure 6:**
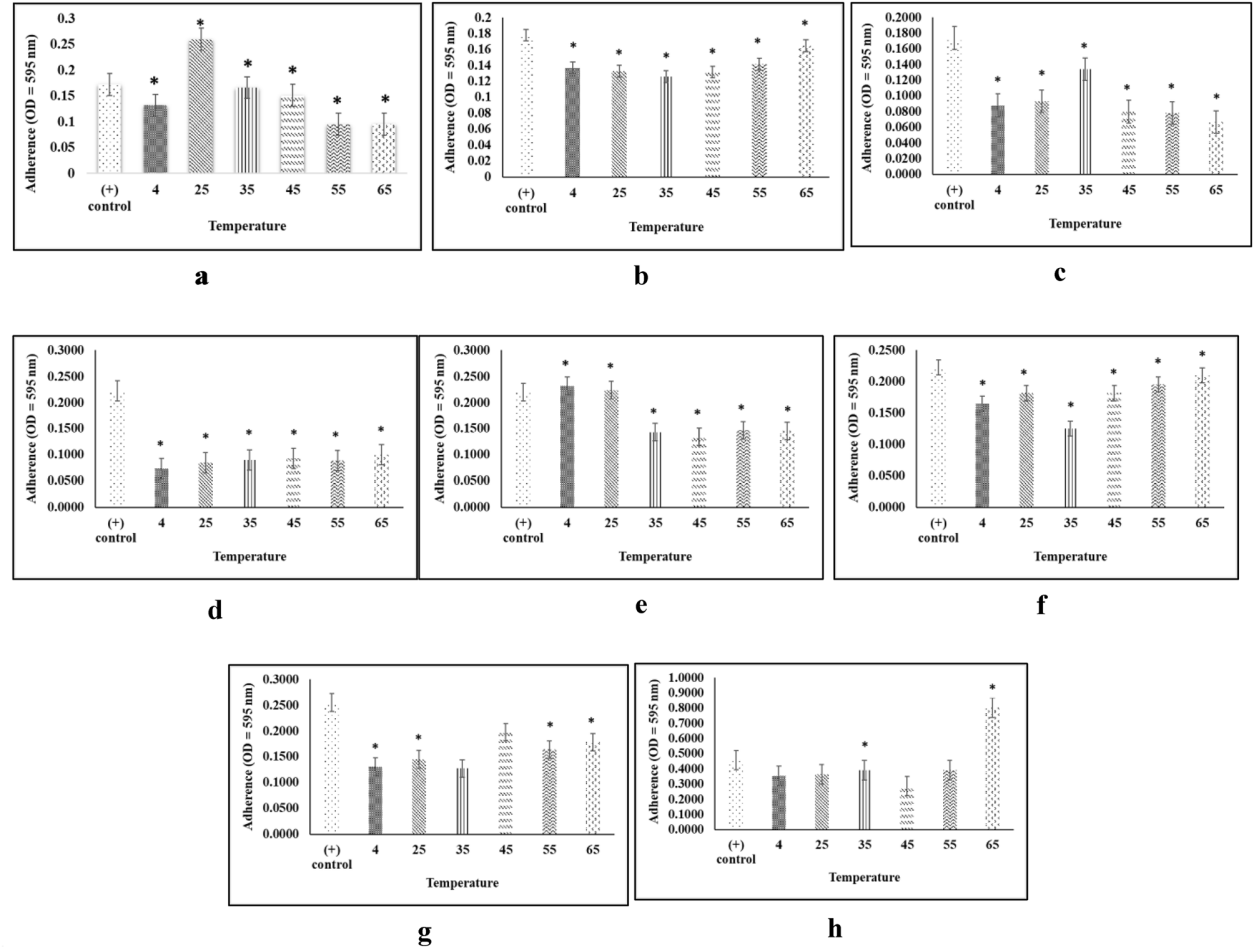
Stability of inhibition activity (**a**) 16PM, (**b**) 20PM, (**c**) 18PM, (**d**) 1AC and destruction activity (**e**) 16PM, (**f**) 20PM, (**g**) 18PM, and (**h**) 1AC (n = 6 replications; vertical bars are standard errors; *: significantly different at p < 0.05).

All of crude extracts were tested for the stability of their biofilm inhibition and destructive activity in the temperature range of 4–65 °C (Fig. 6). The inhibition activity of the 16 PM extract was found unstable at 25 °C (Fig. 6a), and the inhibition activities of 20 PM, 18 PM, and 1 AC extracts were stable at 4–65 °C (Fig. 6b–d). Similarly, the destructive activity of the 16 PM extract was unstable at 4 and 25 °C (Fig. 6e), but the destructive activities of 20 PM and 18 PM extract were stable at 4–65 °C (Fig. 6f, g). The destructive activity of 1 AC extract was unstable at 65 °C (Fig. 6h).

### Antibiofilm activity on stainless steel coupons

All of crude extracts showed inhibition and destruction activity against biofilms formed by foodborne and fish pathogens on stainless steel coupons (see Supplementary Fig. S1 online).

### Genetic identification of *Actinomycetes* isolates

The four *Actinomycetes* isolates showed high similarity to *Streptomyces*. The sequences were submitted to Genbank under accession numbers MW680902 (16 PM), MW680905 (20 PM), MW680906 (18 PM), and MW680936 (1 AC) (Supplementary Table S1).

### SEM observation of biofilm structure

**Figure 7:**
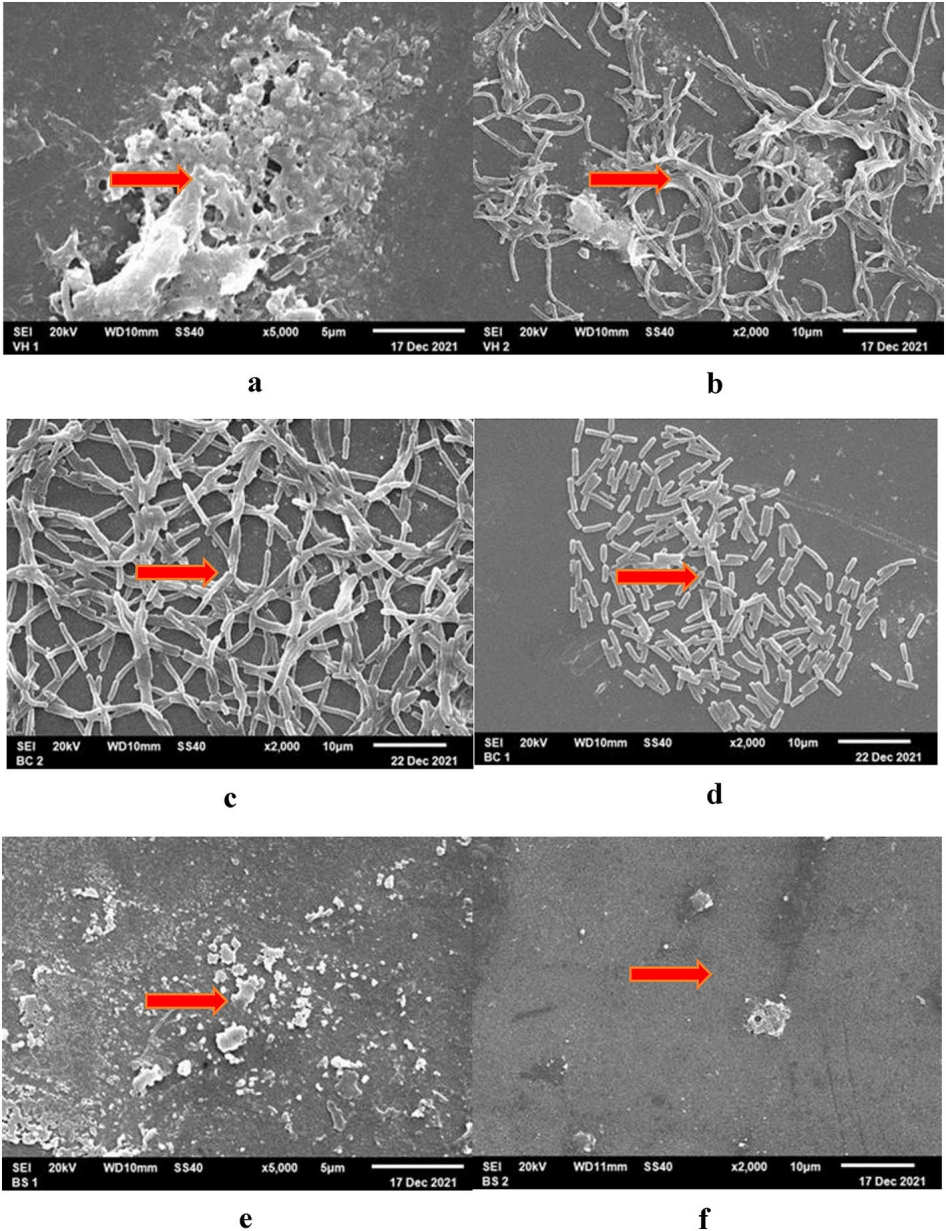
The biofilm structure of *V. harveyi* (**a**) positive control, (**b**) treated with crude extract of 20 PM isolate; *B. cereus* (**c**) positive control, (**d**) treated with crude extract of 1AC isolate; and *B. subtilis* (**e**) positive control, (**f**) treated with crude extract of 18 PM isolate.

The biofilm structure after treated with crude extract were observed with SEM (Fig. 7).

### EDS identification of elemental composition

EDS data showed that no elements were lost or added after treating *V. harveyi* biofilm with 20 PM crude extract. However, aluminum was lost after treating *B. cereus* bacteria with 1 AC crude extract, and carbon was lost after treating *B. subtilis* bacteria with 18 PM crude extract (see Supplementary Fig. S2 online and Supplementary Table S2).

### Phylogenetic tree of *Actinomycetes* isolates

**Figure 8:**
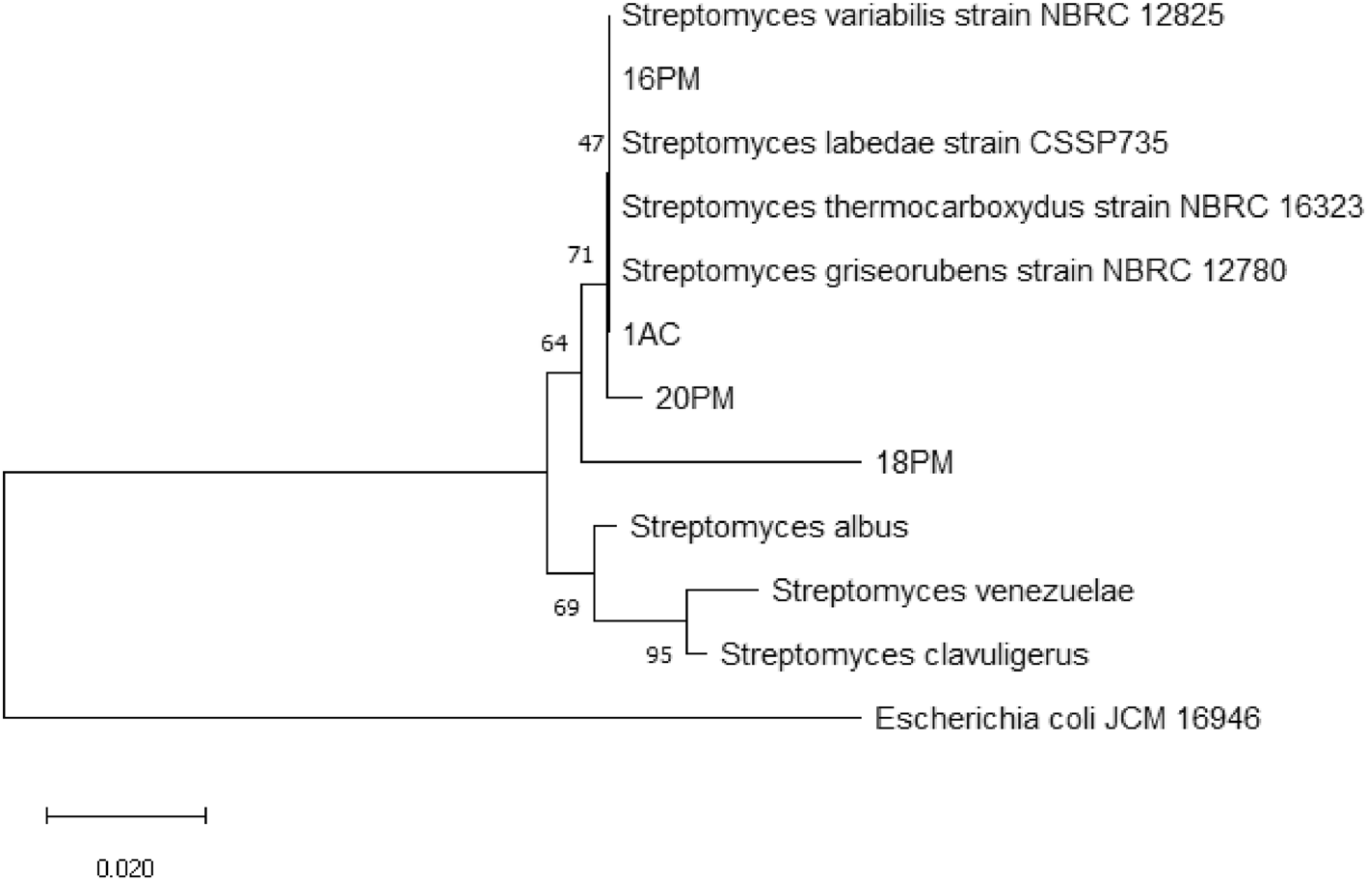
Phylogenetic tree of *Streptomyces* based on the 16S rRNA gene sequence. The bar indicates a distance of 0.020 substitutions per site.

Phylogenetic analysis of *Actinomycetes* isolates based on 16S rRNA gene sequences showed that the 16 PM isolate was closely related to 20 PM and 1 AC. The 18 PM isolate demonstrated a slightly more distant kinship from the other isolates (Fig. 8).

### GC–MS analysis

GC–MS analysis showed that the 16 PM, 18 PM, and 1 AC ethyl acetate crude *Actinomycete* extracts contained 22 constituents, 31 constituents, and 19 constituents, respectively (see Supplementary Fig. S3 online and Supplementary Table S3). The following major compounds present in the 16 PM crude extract were identified from some of the highest peaks: (1) coniine/(S)-2-propylpiperidine, with a retention time of 11.210; (2) pyrene, with a retention time of 16.949; (3) 4,8-dihydroxy-2-(1′-hydroxyheptyl)-3,4,5,6,7,8-hexahydro-2H-[1]-benzopyran-5…, with a retention time of 20.731. The major compounds in the 18 PM crude extract were (1) piperidine, 2-(tetrahydro-2-furanyl), with a retention time of 14.44; (2) 1,3-cyclohexanedione, 2,5,5-trimethyl-(CAS)/2-methyldimedone with a retention time of 20.777; (3) pyrrolo[1,2-a]pyrazine-1,4-dione, hexahydro-3-(phenylmethyl), with a retention time of 17.982. Finally, the major compounds present in 1 AC crude extract were (1) coniine/(S)-2-propylpiperidine, with a retention time of 11.301; (2) 2-(3′-hydroxypropyl)-3,5,6-trimethyl-1,4-benzoquinone, with a retention time of 16.992; (3) phenol, 2,6-dimethoxy-(CAS)/2,6-dimethoxyphenol/dimethoxy phenol, with a retention time of 20.773.

## Discussions

Although polysaccharides are known as one of the most common antibiofilm agents, protein and extracellular DNA (eDNA) also demonstrate antibiofilm activity, effectively inhibiting or destroying biofilms^13^. In this study, we characterized the bioactive compounds of crude actinomycete extracts by treating them with protease, DNAse, and amylase and determining their post-treatment antibiofilm properties. DNase degrades the extracellular DNA (eDNA) in the matrix, weakening it and making it susceptible to antibiofilm activity^14^. Protease degrades proteins, resulting in bacterial cell lysis and growth inhibition^15^. Amylase hydrolyzes the polysaccharide backbone of extracellular polymeric substances (EPS)^16^. The antibiofilm activity of 18 PM and 1 AC extracts against *Shewanella putrefaciens* biofilm decreased amylase treatment (Figs. 3b, 4b). In addition, we discovered that several extracts contained multiple active compound types. For example, 1 AC crude extract used against *B. subtilis* biofilms contained proteins and nucleic acids (Fig. 4g, i). The 20 PM extract used against *V. harveyi* and 18 PM extract used against *B. subtilis* both contained a combination of polysaccharides and nucleic acids (Figs. 2e, f, 3h, i), 18 PM and 1 AC used against *B. cereus* contained a combination of proteins and polysaccharides (Figs. 3d, e, 4d, e), and 16 PM used against *A. hydrophila* and *V. harveyi* contained a combination of the polysaccharides, proteins, and nucleic acids (Figs. 1, 2a–c).

According to the results of the characterization analysis, bacteria from the same genus produce different antibiofilm compounds against different pathogens and do not share the spectrum of antibiofilm activity or the compounds responsible for it, implying that bacteria from Mutiara Beach (16 PM, 20 PM, and 18 PM) and Ancol Beach (1 AC) specifically inhibit biofilm formation. Despite the fact that the majority of antibiofilm bioacive compounds are derived from the three components (polysaccharide, protein, or nucleic acid), some bacteria produce antibiofilm compounds such as fatty acids, rhamnolipids, and sophorolipids^17^. Some bacteria also produce small molecules with antibiofilm activity, such as D-amino acids, aryl rhodanines, and chelators^18^.

A similar previous study found that pre-treating 20 PM extract with NaIO_4_ reduced antibiofilm activity against *A. hydrophila*, indicating that polysaccharides were the active antibiofilm components^13^. Most antibiofilm polysaccharides function as surfactant molecules, altering the physical properties of bacteria and abiotic surfaces. Moreover, polysaccharides may act as signaling molecules, modulating gene expression in recipient bacteria^19^. Antibiofilm polysaccharides may inhibit lectins or sugar-binding proteins, fimbriae, and pili tip adhesins on bacterial surfaces^20^.

Proteins have also exhibited antibiofilm activity. For example, *Actinomycetes* strains use extracellular protease to prevent other bacteria from forming biofilms. Other bacteria reduce biofilm formation to move to different locations and exploit other nutritional sources^21^. Previous research found that *Actinomycetes* with high protease activity reduce *Staphylococcus aureus* biofilm formation and increase biofilm dispersal. Notably, the protein did not affect cell growth and decreased the likelihood of resistance^22,23^. Researchers have also observed that proteins from some *Actinomycetes* strains may inhibit *Staphylococcus epidermidis* biofilm formation by affecting *S. epidermidis* cell adherence^24^.

We found in the current study that extracellular DNA (eDNA) can also demonstrate antibiofilm activity. eDNA is responsible for single-cell attachment and therefore impacts biofilm growth in any stage of biofilm maturation, but it cannot disrupt existing biofilms. eDNA binds specifically to adhesive^25^ and can disrupt the biofilm matrix, eradicating a bacterial colony^26^.

Figure 5 showed the pH profile of the crude extracts of antibiofilm activity. The inhibitory and destructive antibiofilm activity was the highest at pH 6. Similarly, a previous study found that the optimum pH for bioactive compound production for *Streptomyces* strains is near neutral^27^. Regarding thermal stability, the 16 PM extract did not maintain biofilm inhibition activity at 25 °C (Fig. 6a), but the 20 PM, 18 PM, and 1 AC extracts performed consistently at 4–65 °C (Fig. 6b–d). For biofilm destruction, the 16 PM extract was unstable at 4 °C and 25 °C (Fig. 6e), but 20 PM and 18 PM extracts were stable at 4–65 °C (Fig. 6f, g). Moreover, at 65 °C, the 1 AC extract was found to be unstable (Fig. 6h). The pH and temperature ranges were chosen based on the susceptibility of food to pathogenic bacterial growth, which occurs on average at pH 3–10 and a temperature of 4–66 °C^28,29^. Furthermore, optimum growth conditions for the fish pathogen *A. hydrophila* are pH 4.5–7.2 and 5–45 °C^30^. Using heat- and pH-resistant antibiofilm compounds such as the ones we identified, it will provides an advantage in pathogen treatment for both aquaculture and in food processing application. Heat or pH alone, however, cannot completely kill the pathogen.

We investigated the antibiofilm activity of four *Actinomycetes* extracts against *A. hydrophila* (16 PM and 20 PM) and *B. subtilis* (18 PM and 1 AC) on stainless steel coupons to determine if the active components of crude *Actinomycete* extracts could still combat biofilms formed on stainless steel material commonly used in industrial instruments. All of crude extracts demonstrated inhibition and destruction of biofilms of foodborne and fish pathogens on the coupons (see Supplementary Fig. S1 online).

The *Actinomycete* isolates were determined to be within the genus *Streptomyces* using amplification of the 16S rRNA gene (Supplementary Table S1). The genus is distinguished by the production of a wide range of extracellular enzymes and bioactive secondary metabolites with diverse structural and functional properties that are used as antibacterial, antifungal, antiviral, antioxidant, immunomodulatory, anticancer, antibiofilm agents^12^, and anti-vibrio compounds^31–34^*. Streptomyces labedae,* for example, has been shown to inhibit the biofilm-forming ability of *Acinetobacter* and *Moraxella*^35^, and *S. variabilis* has an antibiofilm effect against the human clinical pathogens *V. cholerae, Escherichia coli,* and *Staphylococcus aureus*^36^*.*

We used SEM imaging to determine the structure of biofilms exposed to the tested antibiofilm extracts. The biofilms exposed to 18 PM and 1 AC crude extracts were significantly different from the control (Fig. 7d, f), and *B. subtilis* and *B. cereus* bacterial cells were largely destroyed by 18 PM and 1 AC crude extracts, respectively. The biofilms exposed to the 20 PM extract were similar to untreated cells (Fig. 7b).

We also employed EDS to determine the elemental content of positive control and crude extract-treated biofilms (Supplementary Table S2). Before treatment with 20 PM crude extract, the EDS analysis of *V. harveyi* biofilm structure revealed the following % weight composition: 20.00% carbon (C), 32.23% oxygen (O), 5.86% sodium (Na), 2.01% magnesium (Mg), 34.54% silicon (Si), and 5.36% calcium (Ca). After treatment with 20 PM crude extract, the % weight biofilm composition of *V. harveyi* was 39.56% C, 23.18% O, 2.98% Na, 1.37% Mg, 28.4% Si, and 4.51% Ca. The % weight of C was higher in the treated biofilms than in the positive control, while the % weight of elements such as O, Na, Mg, Si, and Ca was lower. The EDS results were consistent with the SEM results (Fig. 7b), which showed that the biofilm structure was disintegrating, but many bacterial cells were still attached. The subsequent antibiofilm activity assay revealed that the 20 PM crude extract destroyed 65.90% of the *V. harveyi* biofilm. Moreover, the 18 PM and 1 AC destroyed 71.55% and 70.54% of the *B. cereus* and *B. subtilis* biofilms, respectively.

EDS analysis revealed that the *B. cereus* positive control % weight composition was 44.84% C, 23.65% O, 3.37% Na, 1.14% Mg, 23.05% Si, 3.33% Ca, and 0.63% Al. After treatment with 1 AC crude extract, the % weight composition was 18.10% C, 31.24% O, 4.90% Na, 1.92% Mg, 37.80% Si, 6.04% Ca, and Al was not detected. This result suggests that the % weight of C decreased substantially after treatment, while the % weight of other elements such as O, Na, Mg, Si, and Ca increased. The biofilm structure of *B. cereus* indicated that the bacterial cells were less attached to each other (Fig. 7d). This result could be due to a lack of Al elements after treatment. As biofilms grow, Al promotes an increase in the biofilm mass and improves biofilm activity. Al has previously been found to benefit proliferation, stimulating microorganisms to form larger colonies for mature biofilms and increasing EPS protein during the early stages of biofilm growth^37^.

Finally, before treatment, the EDS analysis of *B. subtilis* biofilm structure revealed a weight % composition of 20.16% C, 34.33% O, 7.92% Na, 2.57% Mg, 47.77% Si, and 7.40% Ca. After treatment with 18 PM crude extract, the biofilm structure consisted of 32.53% O, 6.02% Na, 1.86% Mg, 33.96% Si, and 5.47% Ca. The EDS result was consistent with the SEM results (Fig. 7f), in which the 18 PM crude extract was observed to reduce the biofilm structure of *B*. *subtilis* significantly.

In the EDS spectra, high levels of silicon were observed for all positive control bacteria and after treatment with the crude extract (Supplementary Fig. S2 online). Silicon promotes biofilm formation on glass surfaces and was previously revealed to be extremely susceptible to pathogen colonization. Silicon may have been adsorbed onto the biofilm growing on the cover glass used in the experiment, and the silicon peak confirmed this adsorption^38^.

Semi-quantitative EDS data revealed that specific elements might be important for biofilm formation. Magnesium is necessary for bacterial metabolism, directly affecting cell multiplication rates of various species. Magnesium deficiency can cause the ribonucleoprotein complex to degrade and is linked to cell viability loss^38^. In a previous study, magnesium was found to increase *Pseudomonas fluorescens* bacterial adhesion, surface colonization, and biofilm depth but did not affect planktonic cell growth^39^.

Present in a wide range of substrates, carbon is crucial because most pathogenic bacteria are heterotrophic and use carbon as an energy source^38^. Oxygen is also important, as the size and architecture of biofilms may be affected by oxygen availability. Moreover, some bacteria cannot form biofilms in the absence of oxygen. A previous study found that a lack of oxygen may be a detachment signal for *E. coli* biofilms^40^, and it has been proposed that oxygen limitations negatively affect the adhesion of the K-12 strain of *E. coli*^41^.

We also measured relative calcium content. Calcium is thought to promote biofilm formation by cross-linking EPS via uronic acid in bacteria, particularly Gram-negative bacteria, improving the biofilm’s mechanical stability^42,43^. Moreover, Ca^2+^ as a divalent cation can link eDNA by connecting negatively charged bacterial surfaces. It has been demonstrated that eDNA at the cell surface can bind to calcium and inhibit bacterial aggregation in various bacteria, including *Haemophilus influenzae*^44^ and *Xylella fastidiosa*^45^. Calcium also can interact with biofilm-associated proteins. Many bacteria, including *Pseudomonas putida*, *Salmonella enteritidis*, and *P. fluorescens*, have biofilm-associated surface proteins required for early-stage biofilm formation^46^.

We constructed a phylogenetic tree using the neighbor-joining tree method to determine the relationship between the *Actinomycetes* isolates. This method chooses a sequence combination that provides the most accurate estimate of the closest branch length^47^. To estimate the confidence level of a phylogenetic tree, we used a bootstrap of 1000 replicates. The results of phylogenetic analysis (Fig. 8) show that 16 PM, 20 PM, and 1 AC isolates were closely related. The 18 PM isolate had a slightly more distant relationship. However, the four isolates formed very close groups in the phylogenetic tree.

GC–MS analysis of the crude extracts revealed that coniine/(S)-2-propylpiperidine was the highest active fraction of 16 PM and 1 AC crude extract. Coniine, also known as cicutine, 2-propylpiperidine, and conicine, is an alkaloid found in a wide range of plants, including monocots (*Aloe*) and dicots (*Conium* and *Sarracenia*)^48^. Unfortunately, the literature on microbial alkaloid biochemistry is still limited compared to plant alkaloids. The antibiofilm effects of natural products such as alkaloids are primarily based on the inhibition of polymer matrix formation, the suppression of cell adhesion and attachment, the interruption of extracellular matrix generation, and the decrease of virulence factor production, blocking quorum sensing and biofilm development^49^.

Many studies have found alkaloids to be effective antibiofilm agents. For example, the alkaloid norbgugaine has been shown to significantly affect *P. aeruginosa* biofilm by preventing adhesion due to cell motility loss^50^. Moreover, by altering the quorum-sensing system, berberine inhibits biofilm formation in drug-resistant *E. coli* strains^51^. The alkaloid reserpine inhibits the expression of both luxI/luxR-related genes, reducing *P. aeruginosa* biofilm formation via quorum sensing^52^. Total alkaloids of *Sophora alopecuroides*, a Chinese herb, were found to inhibit biofilm formation of field *S. epidermidis* isolated from a cow with mastitis^53^. Moreover, by disrupting the signals from the rhl system, piperidine alkaloid solenopsin A, an alkaloid from the ant *Solenopsis invictan*, was found to inhibit *P. aeruginosa* pyocyanin production and reduce biofilm formation^54^.

The second active compound found in 16 PM crude extract was 4,8-dihydroxy-2-(1′-hydroxyheptyl)-3,4,5,6,7,8-hexahydro-2H-[1]-benzopyran-5 (koninginin D). These compounds have demonstrated potent antibacterial activity against *Acinetobacter baumanii* and *S. aureus*^55^. Pyrene, or 1,2,3,6,7,8-hexahydro-S/1,2,3,6,7,8-hexahydropyrene, was the third active fraction. A polycyclic aromatic hydrocarbon, pyrene is a common pollutant^56^. Unfortunately, no research has been investigated pyrene as an anti-biofilm agent.

Piperidine, 2-(tetrahydro-2-furanyl), was found the most abundant compound in the 18 PM crude extract. It is a saturated heterocyclic secondary amine with antimicrobial, anti-inflammatory, antiviral, and antioxidant activities^57^. Piperidine was previously investigated for its antimicrobial properties in the treatment of enteric pathogen infection and found to prevent *S. typhimurium* invasion into intestinal epithelium models by nearly 95%^58^.

Pyrrolo[1,2-a]pyrazine-1,4-dione, hexahydro-3-(phenylmethyl) was the second active compound isolated from 18 PM crude extract. A previous study found that this compound, derived from *Streptomyces* sp. VITPK9, exhibited anticandidal activity against *Candida albicans*, *C. krusei*, and *C. tropicalis*. Moreover, similar to this molecule, 3-benzyl-hexahydro-pyrrolo[1,2-a]pyrazine-1,4-dione demonstrated significant anti-quorum sensing activity against *P. aeruginosa* PAO1 and *P. aeruginosa* PAH by preventing biofilm formation without inhibiting cell growth within the biofilm, changing the topography and architecture of the biofilm, preventing bacterial adherence, and further biofilm development^59^. Cyclohexane-1,3-dione was discovered to be another active compound found in 18 PM crude extract. Herbicidal, pesticide, antibacterial, anti-inflammatory, anti-tumor, analgesic, anti-convulsant, antiviral, anti-plasmodial, anti-malarial, anti-allergic, anticancer, and other biological activities have been demonstrated from this compound^60^.

2-(3′-Hydroxypropyl)-3,5,6-trimethyl-1,4-benzoquinone and phenol were also active compounds in 1 AC crude extract. A previous study found that AA-861, a benzoquinone derivative, effectively inhibits the formation of *B. subtilis* biofilms by inhibiting the polymerization of TasA amyloid-like fibers^61^. Furthermore, essential oils containing phenols (e.g., thymol, carvacrol, and eugenol) have antibiofilm properties. Essential oils have been shown to inhibit biofilm formation in staphylococci and *Pseudomonas*. Moreover, thymol and carvacrol have been demonstrated showed antibiofilm properties against various bacteria, including *Cryptococcus, Salmonella,* staphylococci*, Enterococcus,* and *Escherichia*. Eugenol also demonstrated antibiofilm properties against *Porphyromonas, Salmonella, Escherichia coli*, and *Listeria*^62,63^.

According to our previous unpublished study, the major active compounds from 20 PM crude extract were (1) coniine/(S)-2-propylpiperidine, with a retention time of 11.256; (2) 2-(3′-hydroxypropyl)-3,5,6-trimethyl-1,4-benzoquinone, with a retention time of 16.983; (3) 1-(5-hexenyl)-6-methoxybicyclo[3.3.0]octan-2-one with the retention time 20.766. Unfortunately, no literature has thus far been published regarding the biological activity of the third major compound of 20 PM crude extract.

## Methods

### Bacterial cultivation

*Actinomycetes* isolates used in this research were obtained from previous studies^64^, isolated from Mutiara Beach, North Jakarta (16 PM, 20 PM, and 18 PM) and Ancol Beach, North Jakarta (1 AC). The isolates were grown in Yeast Malt Extract Agar and incubated at 28 °C for approximately seven days. We used *B. cereus* ATCC 10876, *B. subtilis* ATCC 6633, and *S. putrefaciens* ATCC 8071 as foodborne pathogens. *B. cereus* and *B*. *subtilis* were streaked onto Luria–Bertani agar and incubated at 37 °C overnight, and *S. putrefaciens* was incubated at 28 °C for 2–3 days. Fish pathogenic bacteria (*V. harveyi* and *A. hydrophila*) were obtained from Health Aquatic Organism laboratory of Department of Aquaculture, Faculty of Fisheries and Marine Sciences, IPB University. They were streaked onto Luria agar and incubated at 28 °C overnight.

### Extraction of bioactive compounds

*Actinomycetes *isolates were cultivated in 100 mL of Tryptone Soya Broth with 1% glucose and incubated at 28 °C on a rotary shaker at 120 rpm for 7 days. The bacterial cultures were transferred to 50 mL sterile conical tubes and centrifuged at 6900 × *g* for 25 min. The cell-free supernatants were harvested and mixed with an equal volume of ethyl acetate and then incubated in a rotary shaker at 120 rpm overnight. The solvent layer was evaporated in a rotary evaporator and then held in a vacuum oven at 50 °C until all the solvent layers were entirely evaporated. The extracts were then weighed, and DMSO 1% was added to achieve the final concentration of 20 mg/mL. The extracts were stored at − 20 °C until analysis^65^.

### Bioactive compound characterization

We analyzed crude extracts of *Actinomycetes* by static inhibition via treatment with proteinase K (1 mg/mL), DNase I (100 µg/mL), and amylase (1 mg/mL) at 37 °C for 24 h to determine if the extracts’ antibiofilm activity could be reduced by destroying their active compounds. After treatment with these enzymes, the extracts were assessed in the biofilm inhibitory and destructive activity assays. Pre- and post-treatment extract activity was measured and compared according to a previously published method^66^.

### Antibiofilm activity on stainless steel coupons

Stainless steel coupons were soaked in 10% bleach for 24 h, thoroughly rinsed three times with sterile distilled water to remove residual hypochlorite, and dried under laminar flow. The coupons were treated with 70% ethanol, air-dried for 5 min at room temperature, and autoclaved for 15 min at 121 °C. Overnight pathogen cultures (OD_600_ = 0.132) were introduced into a Petri dish containing a sterile stainless-steel coupon and incubated for 24 and 48 h at 28 °C or 37 °C. A coupon in the uninoculated medium was used as a negative control^67^.

Crude extracts (20 mg/mL) were tested against the biofilm formed by the foodborne and fish pathogenic bacteria. The treated coupon was transferred to a new Petri dish containing 3 mL of crude extract (20 mg/mL) and reincubated at the appropriate temperature. Following incubation, the coupons were carefully removed from the growth medium with sterile forceps, gently tapped to remove excess liquid, and rinsed three times with sterile water to remove loosely adhering bacteria. The coupon was air-dried for 2 min before staining for 15 min with a 0.5% (w/v) crystal violet solution, washed three times with distilled water, and air-dried. The dye bound to the biofilm was then dissolved in 33% glacial acetic acid and measured with a spectrophotometer at 590 nm. Data were presented as the average of three independent trials^68^.

### pH and thermal stability assay

This assay aimed to determine bioactive compound stability in the crude *Actinomycetes* extracts. pH buffers (2, 4, 6, 8, and 10) were used to adjust the pH value of the crude extract. The temperature stability of the crude *Actinomycetes* extracts were incubated in a water bath for 24 h at 4 °C, 25 °C, 35 °C, 45 °C, 55 °C, and 65 °C. The post-treatment extracts were tested to determine their ability to inhibit and destroy biofilms^28–30,66^. For biofilm inhibition test, 100 µL of crude extracts and 100 µL of bacterial cultures (OD_600_ = 0.132) were transferred into 96-well microtiter plates then incubated at pathogens’ respective temperatures for 24 h. Meanwhile, for biofilm destruction test, 100 µL of bacterial culture were transferred into 96-well microtiter plates then incubated at pathogens’ respective temperature. After that, 100 µL of crude extracts will be added and incubated at 37 °C for 24 h^69^.

### Genetic identification of *Actinomycetes* isolates

The genomic DNA of the actinomycetes isolates was extracted using the ZymoBIOMICS DNA miniprep kit (Zymo Research). The 16S ribosomal RNA (rRNA) gene was amplified using the conserved forward primer 63F (5ʹ-CAGGCCTAACACATGCAAGTC-3ʹ) and reverse primer 1387R (5ʹ-GGGCGGAWGTGTACAAGGC-3ʹ)^70^. Amplifications were performed using a 25 µl mixture containing 12.5 μL of GoTaq, 1 μL of 63F primer, 1 μL of 1387R primer, 1 μL of DNA template, and 9.5 μL ddH_2_O. The PCR reaction was programmed to preheat at 94 °C for 2 min, followed by 25 cycles of denaturation at 94 °C for 30 s, annealing at 55 °C for 30 s and elongation at 72 °C for 1 min before a final extension of 72 °C for 20 min^71^. The obtained 16S-rRNA sequences were compared to sequences in the NCBI GenBank database using the Basic Alignment Search Tool (BLAST).

### SEM–EDS analysis of biofilms

The samples used in this assay were selected based on their highest biofilm destruction activity. Five mL of each pathogen in solution (OD_600_ = 0.132) was grown on a 1 × 1 cm cover glass and incubated at 28 °C or 37 °C for 24 h to permit biofilm formation. The cover glass was transferred to a new sterile Petri dish and treated with 100 μL of the sample, then re-incubated at 28 °C for 24 h. DMSO (100 μL, 1% v/v) was used as a positive control. For observation by using SEM–EDS, we fixed the biofilm with 2.5% (v/v) of glutaraldehyde and incubated the fixed biofilms at 4 °C overnight. The biofilm was dehydrated with 30%, 50%, 70%, 96%, and 100% (v/v) alcohol for 15 min at each concentration. The biofilm was dried at 37 °C for 10 min^72^.

### Phylogenetic tree of *Actinomycetes* isolates

The phylogenetic tree was constructed using the neighbor-joining method and the MEGA-X application and assessed using bootstrap analysis with 1000 resamplings^73^.

### GC–MS analysis

The active fractions of the crude extracts were identified by GC–MS performed in a private medical laboratory (Laboratorium Kesehatan Daerah Provinsi DKI Jakarta) using GC 7890 B and MS 5977 B. The crude extract was dissolved in ethyl acetate in a ratio of 1: 1 (w/v). Sample injection volume 1 µL, column type Agilent 19091S-433:93.92873 (30 m length, 250 µm diameter, 0.25 µm width). Helium gas (99.999%) was used as carrier gas at a total flow rate of 24 mL/min, with a time of 36 min at an oven temperature of 325 °C. The number of compounds obtained was reflected in the number of peaks on the chromatogram. The compound names found were interpreted based on the mass spectrum data of each peak matched with the GC–MS Pyrolysis database^74^.

### Statistical analysis

The significance of the data was evaluated using ANOVA. All analyses were performed using the Statistical Package for the Social Sciences (SPSS) version 25^75^.

## Conclusions

In characterizing the antibiofilm activity of four *Actinomycetes* extracts, we discovered that several extracts contained a combination of the active antibiofilm compounds, including polysaccharides, nucleic acids, and proteins. Other compound that might contribute to antibiofilm activities were identified using GC–MS which included coniine/(S)-2-propylpiperidine and piperidine, 2-(tetrahydro-2-furanyl). We also determined that the antibiofilm activity of most of the extracts was stable within a range of biologically relevant temperatures and at a near-neutral pH (pH 6). The crude extracts were effective against biofilms formed by foodborne and fish pathogens on stainless steel coupons. Moreover, their antibiofilm activities were confirmed by analyzing treated biofilms using SEM–EDS. We proposed that *Actinomycetes* extracts as potential candidates for antibiofilm agents to be used in the food and aquaculture industries. Further research is needed to purify the bioactive compounds of the crude extracts and identify the compounds from GC–MS results that were able to work synergistically with polysaccharides, nucleic acids or proteins as antibiofilm agents.

## Supplementary Information

Supplementary Information.

## Data Availability

All data deposited in the Genebank is publicly available with Genbank accession number MW680902 (https://www.ncbi.nlm.nih.gov/nuccore/1995723704#feature_MW680902.1), MW680905 (https://www.ncbi.nlm.nih.gov/nuccore/MW680905), MW680906 (https://www.ncbi.nlm.nih.gov/nuccore/MW680906), and MW680936 (https://www.ncbi.nlm.nih.gov/nuccore/MW680936).
