# Supplementary material for: Antibiofilm properties of bioactive compounds from Actinomycetes against foodborne and fish pathogens
Source: Sci Rep. 2022 Nov 3;12:18614. doi: 10.1038/s41598-022-23455-8 (PMC9633603; doi:10.1038/s41598-022-23455-8)
Supplement: Supplementary file 1 — Supplementary Information. [file 41598_2022_23455_MOESM1_ESM.docx]

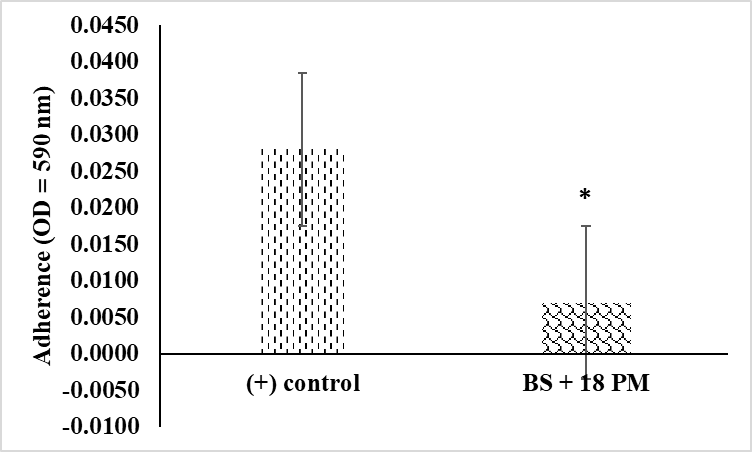

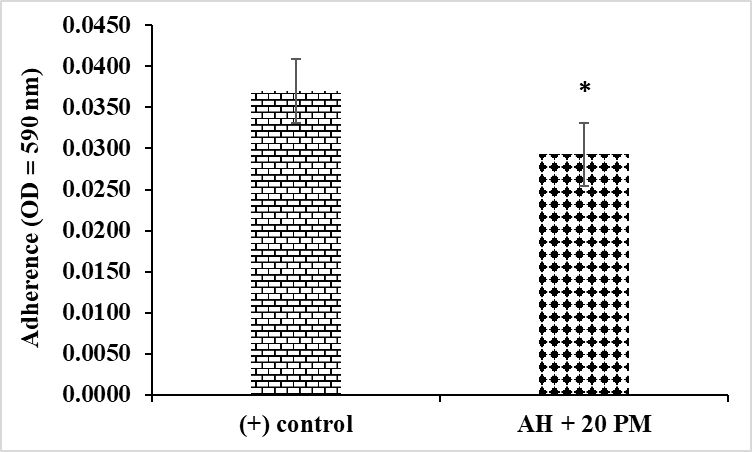

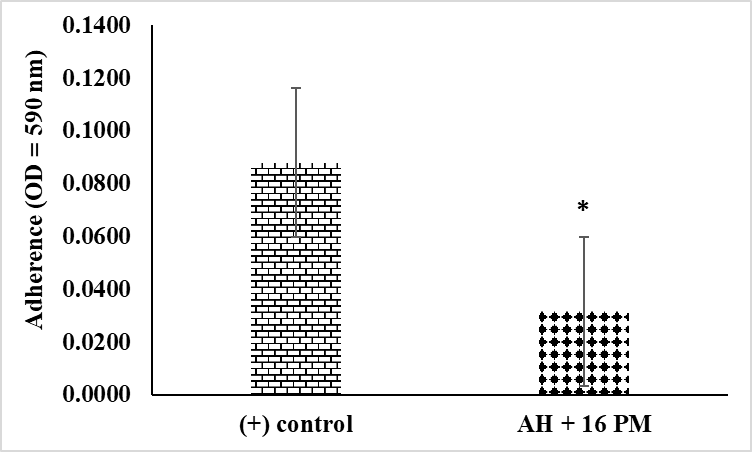
Supplementary Fig. S1

**c**


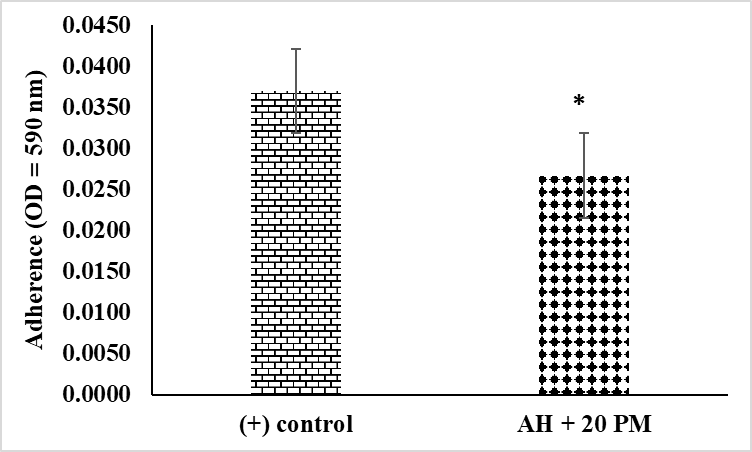


**f**

**b**

**a**


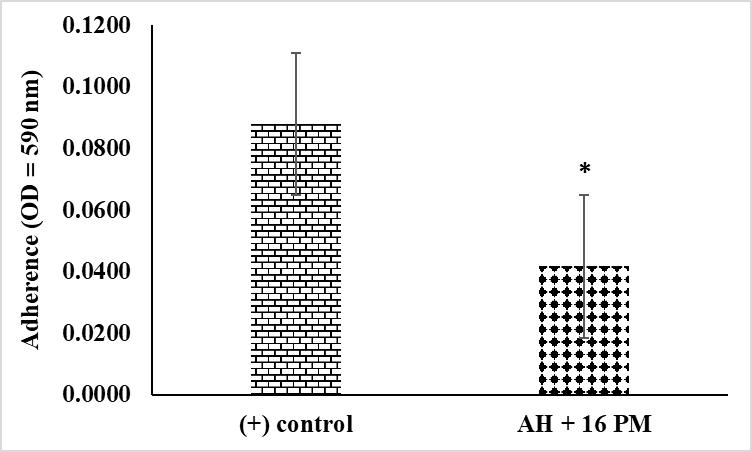

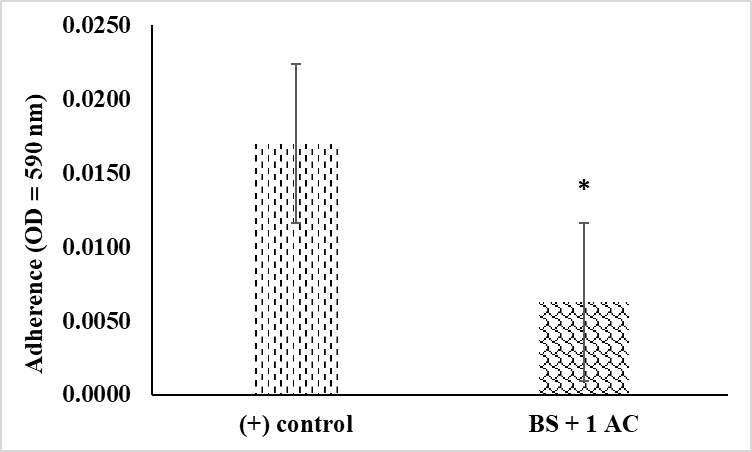


**e**

**d**


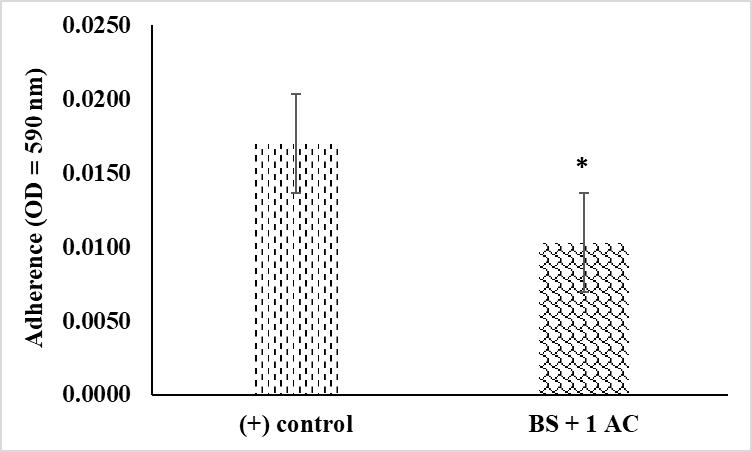

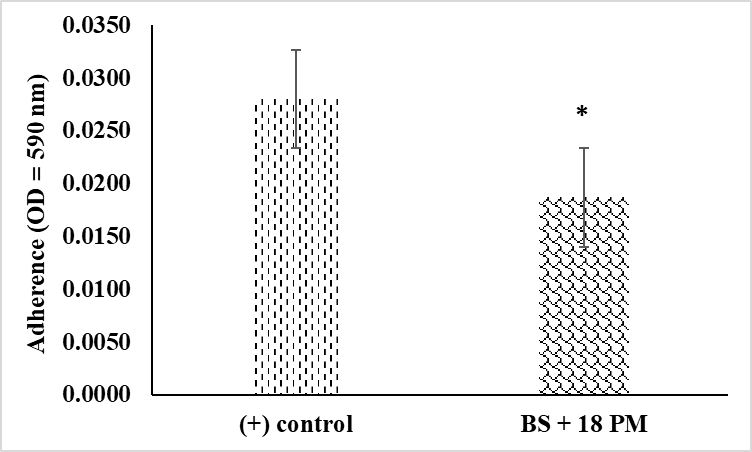


**h**

**g**

Inhibition activity on stainless steel coupon (a) 16PM, (b) 20PM, (c) 18PM, (d) 1AC and destruction activity on stainless steel coupon (e) 16PM, (f) 20PM, (g) 18PM, and (h) 1AC (n = 3 replications; vertical bars are standard errors; *: significantly different at p<0.05). (i) Biofilm formation of *B. substilis* (left) and *A. hydrophyla* (right) on stainless steel coupon.


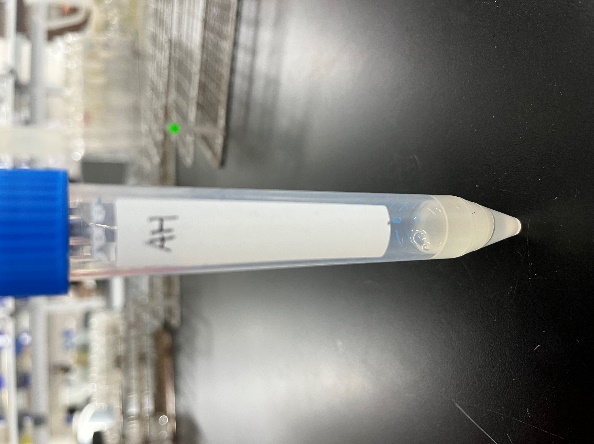


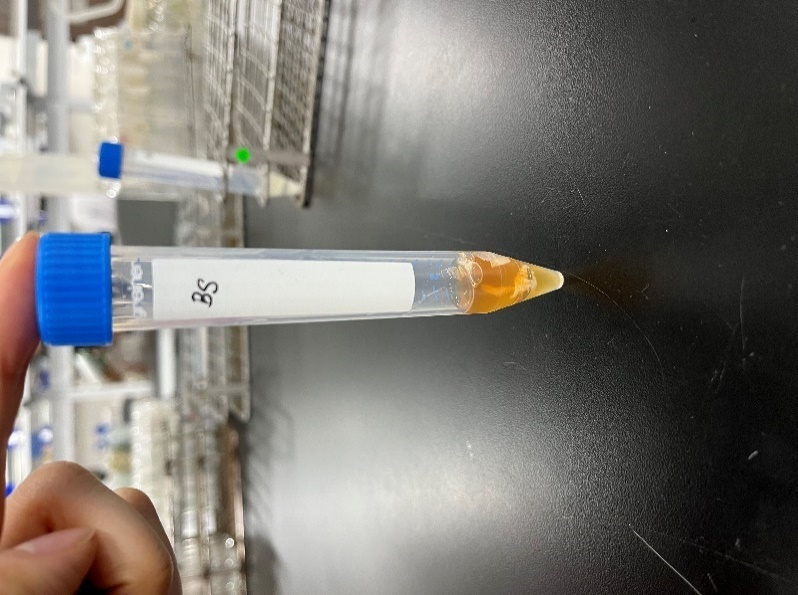


1. Biofilm formation of *B. subtilis* (left) and *A. hydrophila* (right) on stainless steel coupon.

Supplementary Table S1

Sequencing analysis of *Actinomycetes* isolates

|  | | | |
| --- | --- | --- | --- |
| Isolate | Similarity with | % Identity | Accession Number |
| 16PM | *Streptomyces variabilis* NBRC 12825 | 95.44 | MW680902 |
| 20PM | *Streptomyces labedae* CSSP735 | 97.40 | MW680905 |
| 18PM | *Streptomyces thermocarboxydus* NBRC 16323 | 85.22 | MW680906 |
| 1AC | *Streptomyces griseorubens* NBRC 12780 | 99.13 | MW680936 |

Supplementary Fig. S2
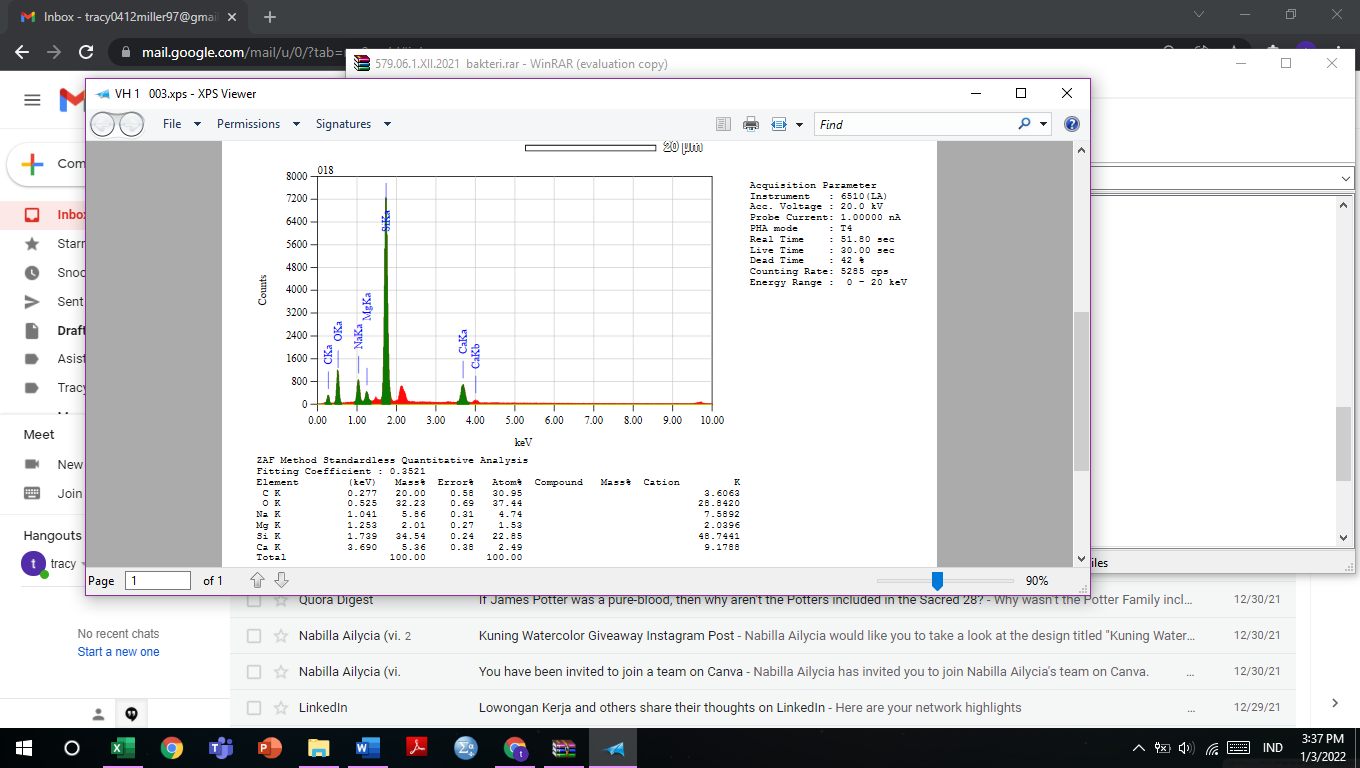

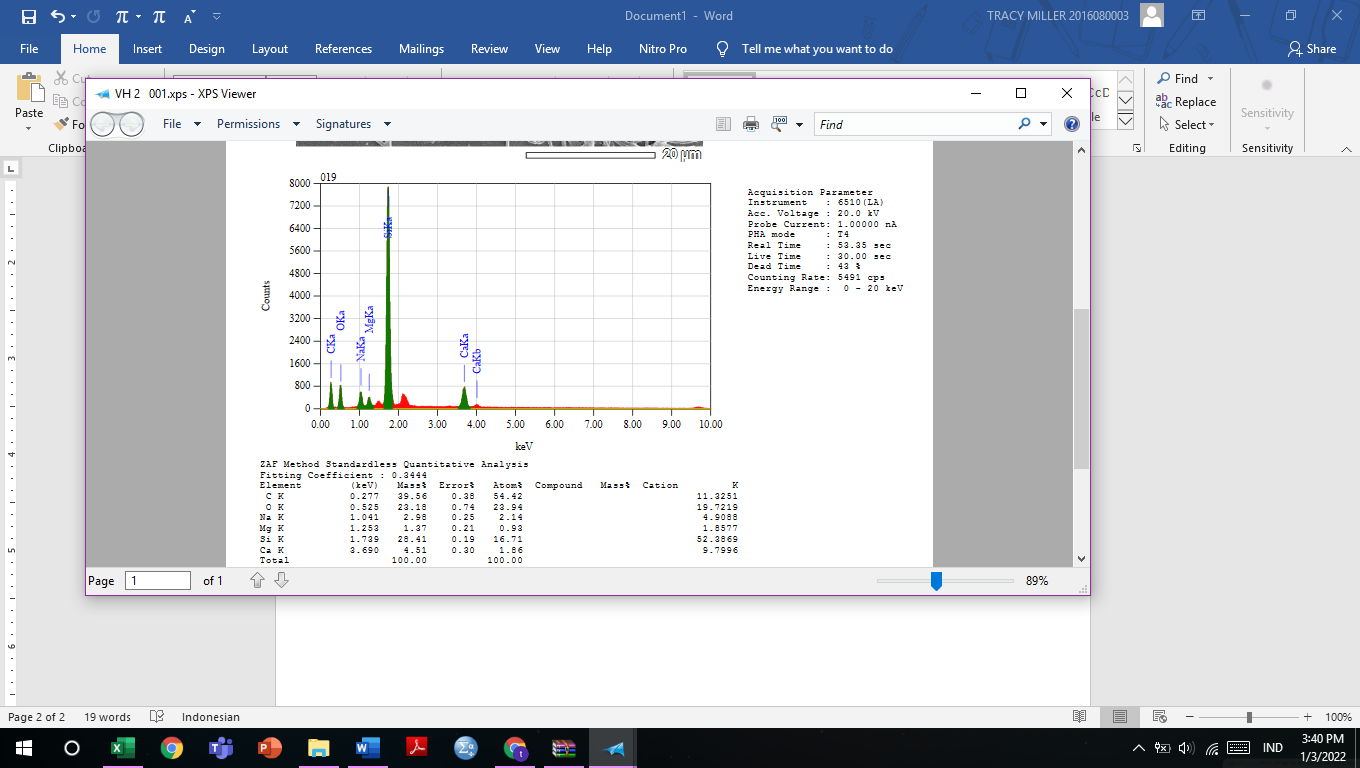


**b**

**a**


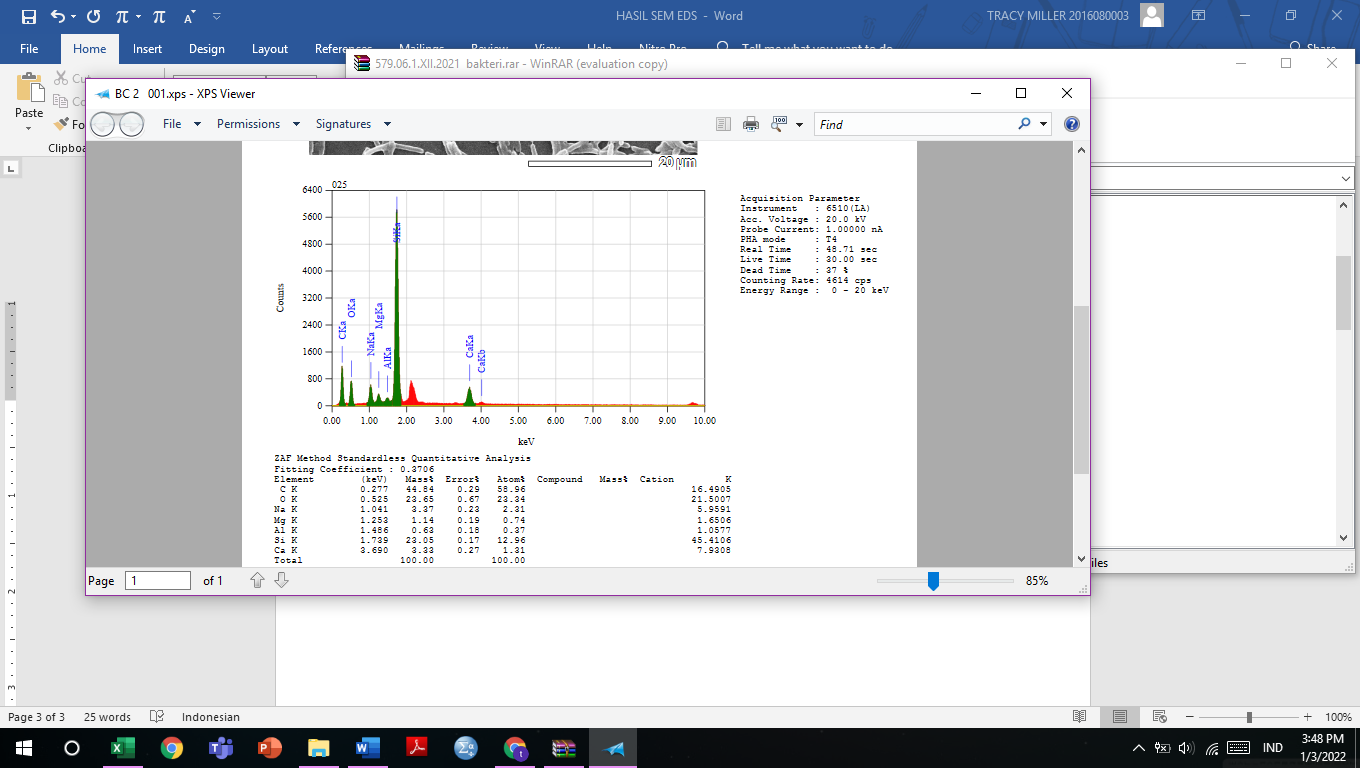

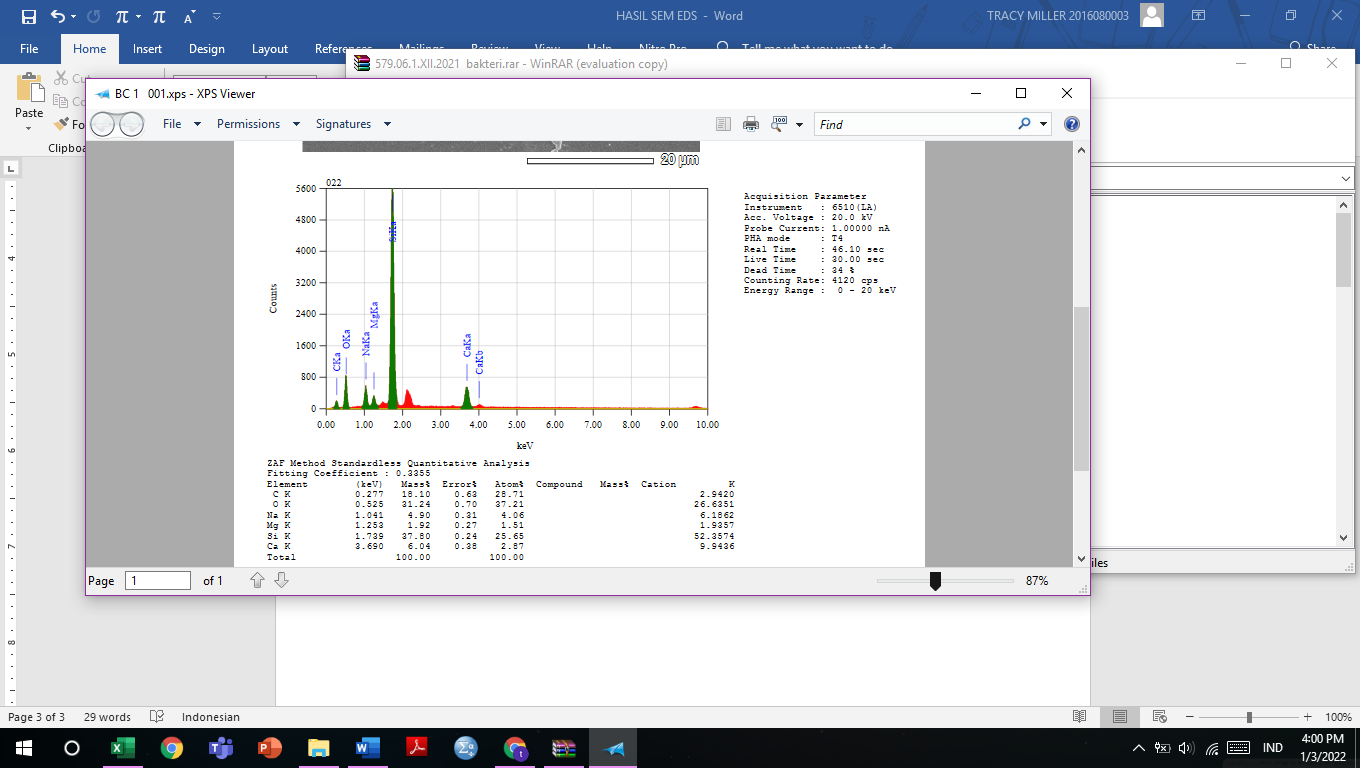


**c**

**d**


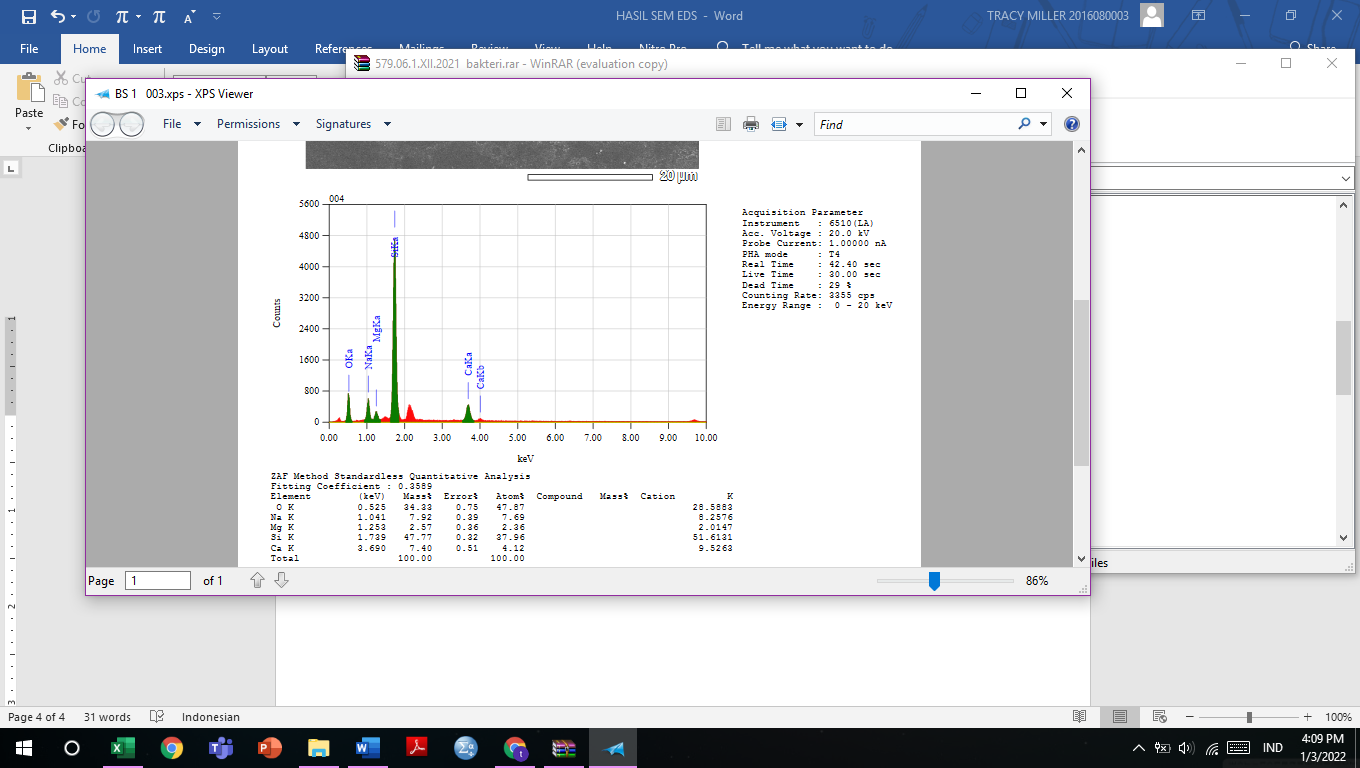

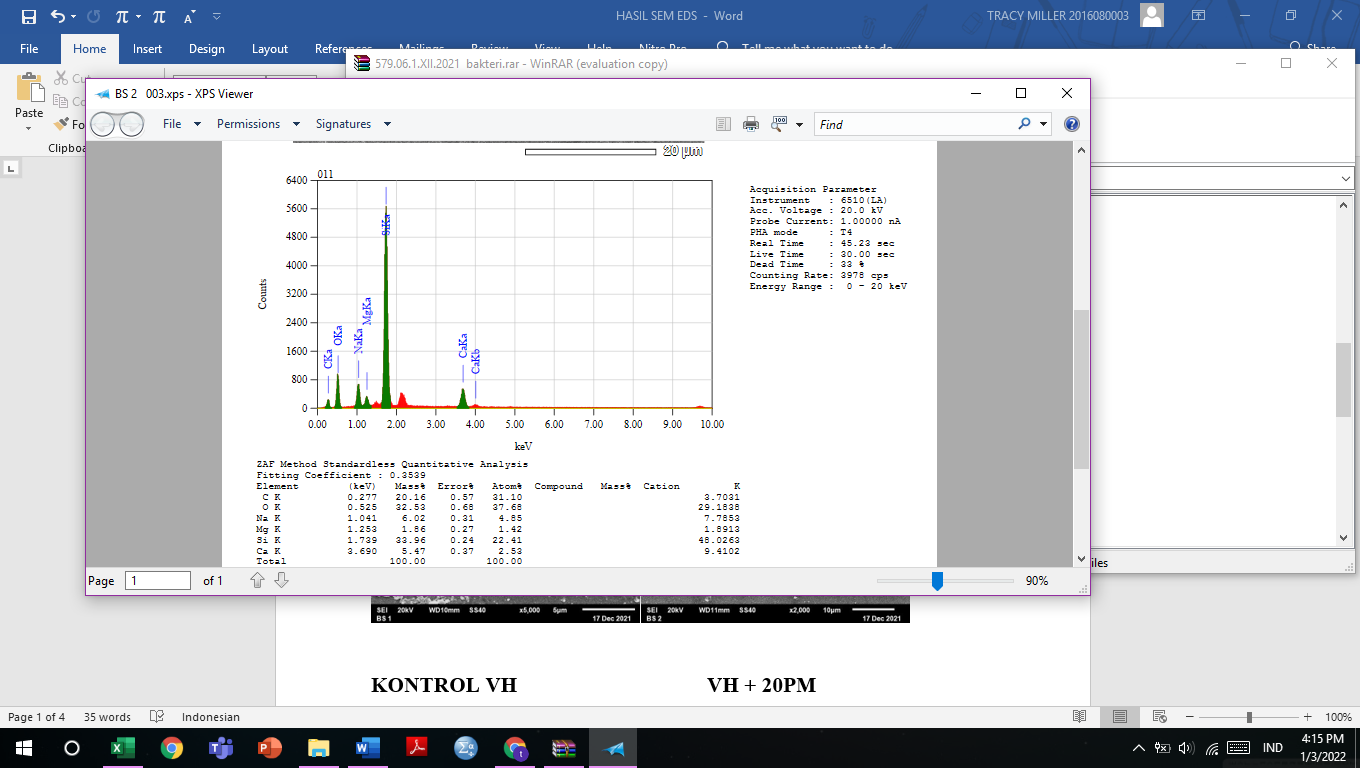


**e**

**f**

The EDS spectra of *V. harveyi* (a) positive control, (b) treated with crude extract of 20 PM isolate; *B. cereus* (c) positive control, (d) treated with crude extract of 1 AC isolate; *B. subtilis* (e) positive control, (f) treated with crude extract of 18 PM isolate

Supplementary Table S2

EDS analysis (weight %) of the biofilm samples (positive control)

| Bacteria | C | O | Na | Mg | Si | Ca | Al |
| --- | --- | --- | --- | --- | --- | --- | --- |
| *V. harveyi* | 20.00 | 32.23 | 5.86 | 2.01 | 34.54 | 5.36 | ND |
| *B. cereus* | 44.84 | 23.65 | 3.37 | 1.14 | 23.05 | 3.33 | 0.63 |
| *B. subtilis* | 20.16 | 34.33 | 7.92 | 2.57 | 47.77 | 7.40 | ND |

EDS analysis (weight %) of the biofilm samples (after crude extract treatment)

| Bacteria | C | O | Na | Mg | Si | Ca | Al |
| --- | --- | --- | --- | --- | --- | --- | --- |
| *V. harveyi* + 20 PM | 39.56 | 23.18 | 2.98 | 1.37 | 28.41 | 4.51 | ND |
| *B. cereus* + 1 AC | 18.10 | 31.24 | 4.90 | 1.92 | 37.80 | 6.04 | ND |
| *B. subtilis* + 18 PM | ND | 32.53 | 6.02 | 1.86 | 33.96 | 5.47 | ND |

Supplementary Fig. S3


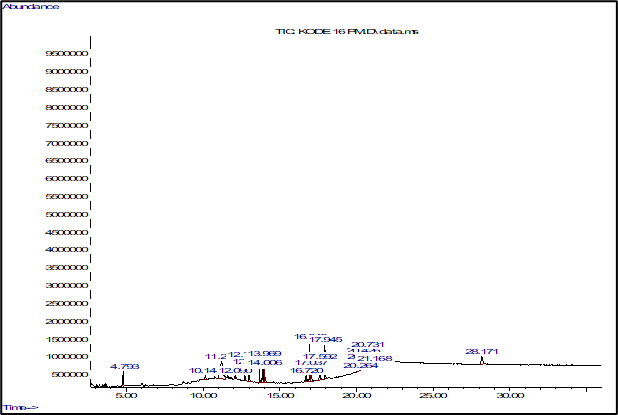


**a**


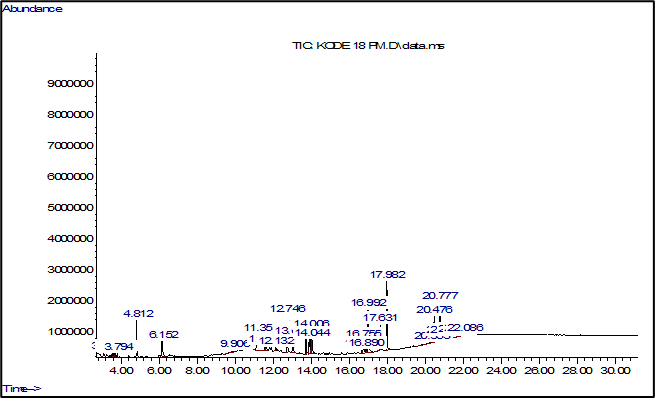


**b**


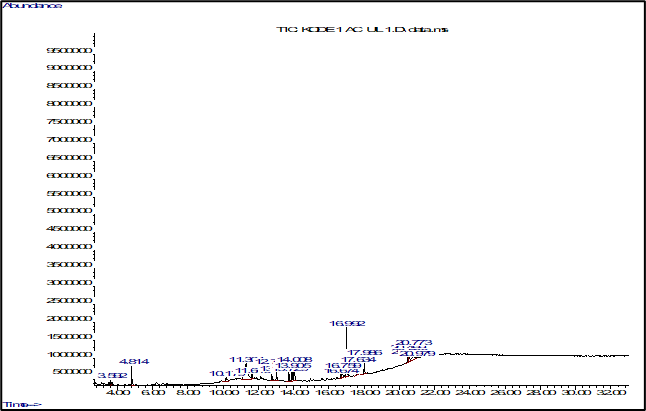


**c**

GC-MS chromatogram of the active fraction from (a) 16 PM crude extract; (b) 18 PM crude extract; (c) 1 AC crude extract.

Supplementary Table S3

GC-MS profile of the active fraction from 16 PM crude extract

| **No** | **Name of the compound** | **Similarity Index** | **Area %** | **Molecular Formula** | **Structure** | **Biological Properties** | **References** |
| --- | --- | --- | --- | --- | --- | --- | --- |
| 1 | 2,3-Dihydro-3,5-dihydroxy-6-methyl-4H-pyran-4-one | 90 | 2.74 | [C_6_H_8_O_4_](https://pubchem.ncbi.nlm.nih.gov/#query=C6H8O4) | 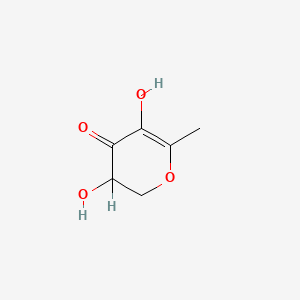 | Antioxidant activity | https://link.springer.com/article/10.1007/s00217-011-1527-4 |
| 2 | 3-Methyl-4-phenylpyrrole | 70 | 1.61 | [C_11_H_11_N](https://pubchem.ncbi.nlm.nih.gov/#query=C11H11N) | 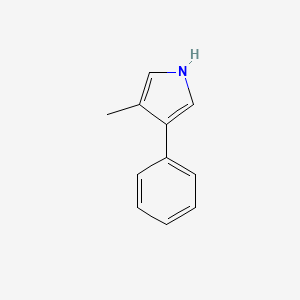 | - | - |
| 3 | Coniine / (S)-2-Propylpiperidine / Piperidine, 2-propyl-, (S)- Cicutin | 72 | 20.06 | [C_8_H_17_N](https://pubchem.ncbi.nlm.nih.gov/#query=C8H17N) | 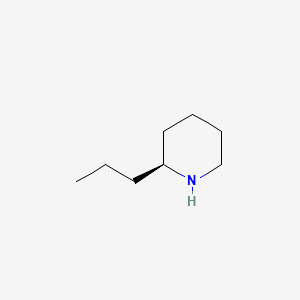 | Neurotoxin, local analgesic in form of coniine salt | <https://link.springer.com/referenceworkentry/10.1007/978-3-642-22144-6_28?noAccess=true> |
| 4 | Pyrrolo[1,2-a]pyrazine-1,4-dione, hexahydro- | 55 | 0.70 | [C_10_H_16_N_2_O_2_](https://pubchem.ncbi.nlm.nih.gov/#query=C10H16N2O2) | 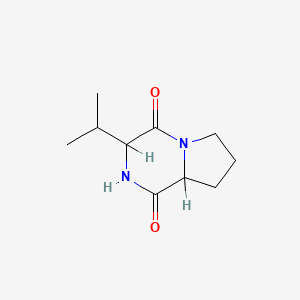 | Algicidal activity | https://link.springer.com/article/10.1007/s00253-014-6043-6 |
| 5 | 1-(5-Hexenyl)-6-methoxybicyclo.0]octan-2-one | 47 | 4.07 | C_14_H_22_O_2_ | 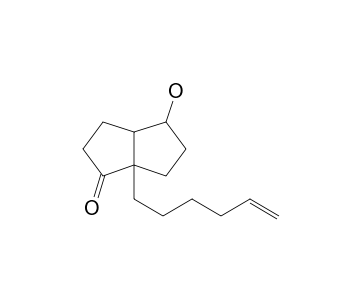 | - | - |
| 6 | Phenol, 3,5-dimethoxy-/ Phloroglucinol dimethyl ether / Taxicatigenin | 50 | 4.81 | [C_8_H_10_O_3_](https://pubchem.ncbi.nlm.nih.gov/#query=C8H10O3) | 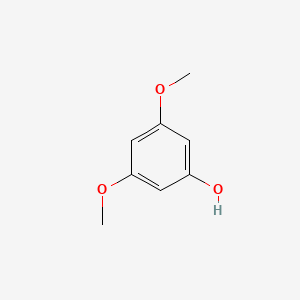 | Potential as antibacterial and antifungal plant material (*Taxus baccata*) | <https://sanitas.e-journal.id/index.php/SANITAS/article/view/20180601-irwandi/20180601-35-43>  <http://jddtonline.info/index.php/jddt/article/view/3635> |
| 7 | Pyrrolo[1,2-a]pyrazine-1,4-dione, hexahydro-3-(2-methylpropyl)- | 91 | 2.37 | C_11_H_18_N_2_O_2_ | 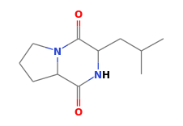 | Antioxidant activity | file:///C:/Users/A450L%20Series/Downloads/Marine_Sp_VITMK1_Derived_Pyrrolo_1_2-A_Pyrazine-1_.pdf |
| 8 | Pyrrolo[1,2-a]pyrazine-1,4-dione, hexahydro-3-(2-methylpropyl)- | 94 | 4.08 | C_11_H_18_N_2_O_2_ | 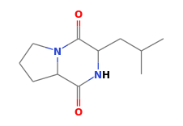 | Antioxidant activity | file:///C:/Users/A450L%20Series/Downloads/Marine_Sp_VITMK1_Derived_Pyrrolo_1_2-A_Pyrazine-1_.pdf |
| 9 | 3,9-DIAZATRICYCLO[7.3.0.0(3,7)]DODECAN-2,8-DIONE / Cyclo-L-prolyl-L-proline | 46 | 4.78 | [C_10_H_14_N_2_O_4_](https://pubchem.ncbi.nlm.nih.gov/#query=C10H14N2O4) | 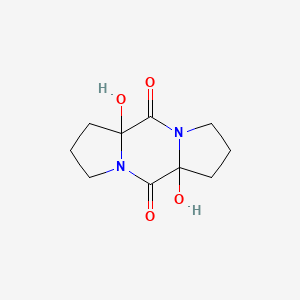 | - | - |
| 10 | Pyrrolo[1,2-a]pyrazine-1,4-dione, hexahydro-3-(2-methylpropyl)- | 80 | 3.73 | C_11_H_18_N_2_O_2_ | 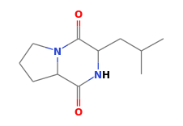 | Antioxidant activity | file:///C:/Users/A450L%20Series/Downloads/Marine_Sp_VITMK1_Derived_Pyrrolo_1_2-A_Pyrazine-1_.pdf |
| 11 | Methyl exo,exo-5,6-dihydroxybicyclo[2.2.1]heptane-exo-2-carboxylate | 50 | 1.98 | - | - | - | - |
| 12 | Pyrene, 1,2,3,6,7,8-hexahydro-S / 1,2,3,6,7,8-Hexahydropyrene | 90 | 9.43 | [C_16_H_16_](https://pubchem.ncbi.nlm.nih.gov/#query=C16H16) | 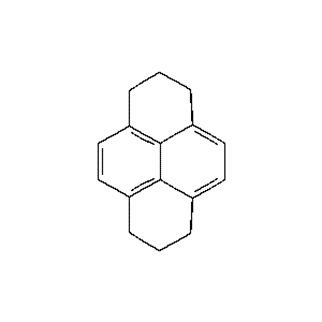 | - | - |
| 13 | 1,8-Diazacyclotetradecane-2 | 64 | 5.18 | - | - | - | - |
| 14 | Ergotamine | 87 | 5.46 | [C_33_H_35_N_5_O_5_](https://pubchem.ncbi.nlm.nih.gov/#query=C33H35N5O5) | 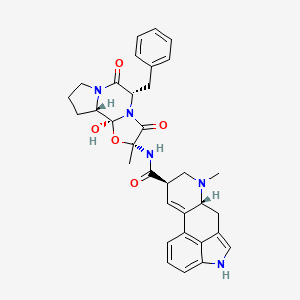 | Acute migraine drug (vasoconstrictor) | https://link.springer.com/referenceworkentry/10.1007/978-3-319-19456-1_1-1?noAccess=true |
| 15 | 3-benzyl-1,4-diaza-2,5-dioxobicyclo[4.3.0]nonane | 98 | 6.86 | C_14_H_20_N_2_ | 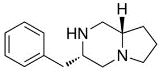 | - | - |
| 16 | 3,5-Dimethyl-2,6-bis(trimethylsiloxy)pyridine | 47 | 0.29 | C_13_H_25_NO_2_Si_2_ | 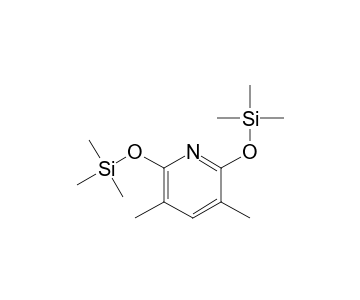 | - | - |
| 17 | 1-(5-Hexenyl)-6-methoxybicyclo[3.3.0]octan-2-one | 47 | 4.44 | C_14_H_22_O_2_ | 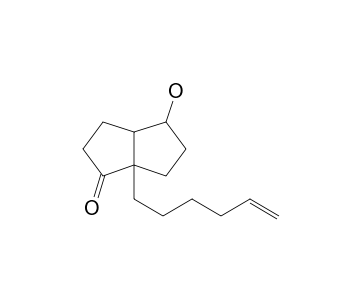 | - | - |
| 18 | Benzo[h]quinoline, 2,4-dimethyl- / 2,4-Dimethylbenzo[h]quinoline | 25 | 3.38 | [C_15_H_13_N](https://pubchem.ncbi.nlm.nih.gov/#query=C15H13N) | 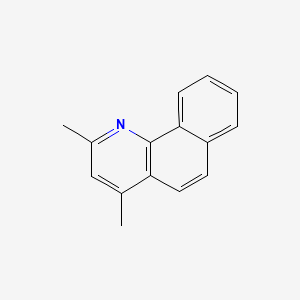 | Plant secondary metabolites | https://link.springer.com/article/10.1007/s11101-019-09649-5 |
| 19 | 4,8-Dihydroxy-2-(1'-hydroxyheptyl)-3,4,5,6,7,8-hexahydro-2H-[1]-benzopyran-5... | 41 | 8.47 | [C_16_H_26_O_5_](https://pubchem.ncbi.nlm.nih.gov/#query=C16H26O5) | 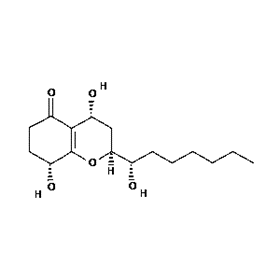 | Antifungal, natural product from the plant endophytic fungus*Trichoderma applanatum* | https://pubmed.ncbi.nlm.nih.gov/29685076/ |
| 20 | 2-Methyl-5H-dibenz[b,f]azepine | 59 | 0.63 | C_15_H_13_N | 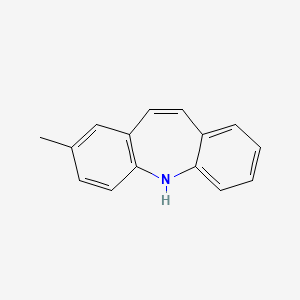 | - | - |
| 21 | 1,2-Bis(trimethylsilyl)benzene / Trimethyl[2 (trimethylsilyl)phenyl]silane | 41 | 0.28 | [C_12_H_22_Si_2_](https://pubchem.ncbi.nlm.nih.gov/#query=C12H22Si2) | 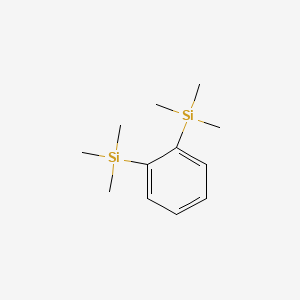 | Hepatoprotective activity | https://link.springer.com/article/10.1007/s42452-021-04859-z |
| 22 | 2-Methyl-5H-dibenz[b,f]azepine | 50 | 4.66 | C_15_H_13_N | 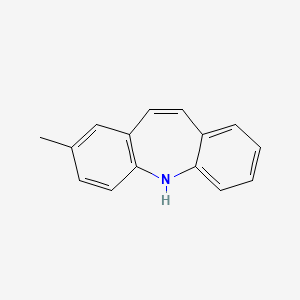 | - | - |

GC-MS profile of the active fraction from 18 PM crude extract

| **No** | **Name of the compound** | **Similarity Index** | **Area %** | **Molecular Formula** | **Structure** | **Biological Properties** | **References** |
| --- | --- | --- | --- | --- | --- | --- | --- |
| 1 | 2,3-Dihydro-3,5-dihydroxy-6-methyl-4H-pyran-4-one | 46 | 0.35 | [C_6_H_8_O_4_](https://pubchem.ncbi.nlm.nih.gov/#query=C6H8O4) | 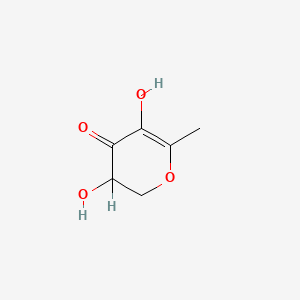 | Antioxidant activity | https://link.springer.com/article/10.1007/s00217-011-1527-4 |
| 2 | 3H-Pyrazol-3-one, 1,2-dihydro-5-methyl / 3-Pyrazolin-5-one, 3-methyl | 58 | 0.34 | [C_4_H_6_N_2_O](https://pubchem.ncbi.nlm.nih.gov/#query=C4H6N2O) | 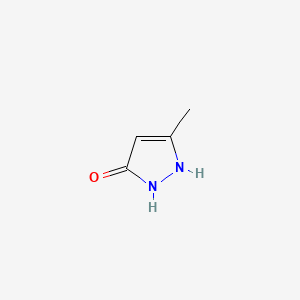 | - | - |
| 3 | PHENYL ACETALDEHYDE / BENZENEACETALDEHYDE / .ALPHA.-TOLUALDEHYDE | 80 | 0.64 | [C_8_H_8_O](https://pubchem.ncbi.nlm.nih.gov/#query=C8H8O) | 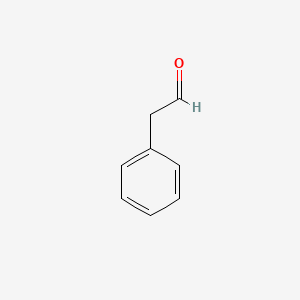 | synthesis of fragrances and polymers. | https://link.springer.com/article/10.1007/s11103-009-9564-0 |
| 4 | Naphthalene (CAS) / White tar / NAPHTALINE / Naphthene / Albocarbon | 58 | 0.32 | [C_10_H_8_](https://pubchem.ncbi.nlm.nih.gov/#query=C10H8) | 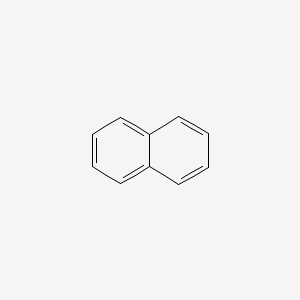 | Naphthalene derivatives have anti-inflammatory activity by inhibiting the activation of neutrophils | https://onlinelibrary.wiley.com/doi/abs/10.1002/ddr.10327 |
| 5 | 4H-Pyran-4-one, 2,3-dihydro-3,5-dihydroxy-6-methyl | 96 | 3.99 | [C_6_H_8_O_4_](https://pubchem.ncbi.nlm.nih.gov/#query=C6H8O4) | 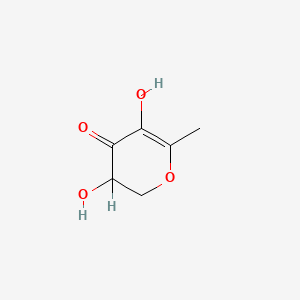 | Antioxidant activity | https://link.springer.com/article/10.1007/s00217-011-1527-4 |
| 6 | Benzeneacetic acid (CAS) / Phenyl acetic acid / 2-PHENYLETHANOIC ACID | 90 | 4.47 | [C_8_H_8_O_2_](https://pubchem.ncbi.nlm.nih.gov/#query=C8H8O2) or C_6_H_5_CH_2_CO_2_H | 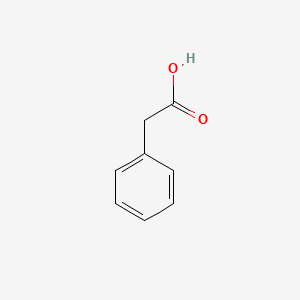 | synthesis of fragrances and polymers. | https://link.springer.com/article/10.1007/s11103-009-9564-0 |
| 7 | 4-[(1Z)-(N-Hydroxyethanimidoyl)-2- methylpyridazin-3(2H)-one | 70 | 0.51 | C_7_H_9_N_3_O_2_ | 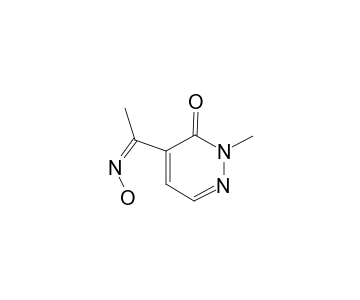 | - | - |
| 8 | Piperidine, 2-(tetrahydro-2-furanyl) | 72 | 14.44 | [C_9_H_17_NO](https://pubchem.ncbi.nlm.nih.gov/#query=C9H17NO) | 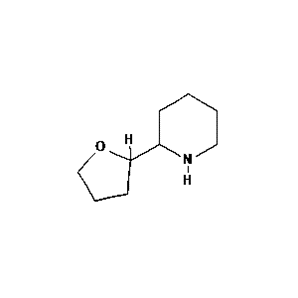 | Local anesthetic, antiseptic agent, sedative, spasmolytic, and hypotensive agent | <https://www.sciencedirect.com/science/article/abs/pii/S0735821099800284> |
| 9 | Cyclopropane, 1,2-dimethyl-1-pentyl / 1,2-Dimethyl-1-pentylcyclopropane | 38 | 1.31 | [C_10_H_20_](https://pubchem.ncbi.nlm.nih.gov/#query=C10H20) | 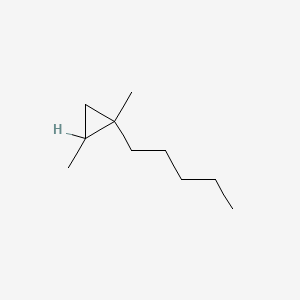 | - | - |
| 10 | Pyrrolo[1,2-a]pyrazine-1,4-dione, hexahydro- | 50 | 0.70 | [C_14_H_16_N_2_O_2_](https://pubchem.ncbi.nlm.nih.gov/#query=C14H16N2O2) | 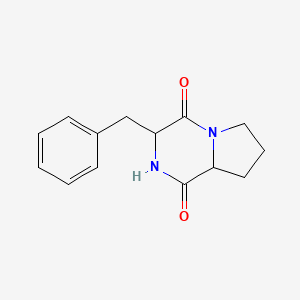 | Algicidal activity | https://link.springer.com/article/10.1007/s00253-014-6043-6 |
| 11 | Cyclo(-L-Pro-L-Val-) | 50 | 4.38 | [C_10_H_16_N_2_O_2_](https://pubchem.ncbi.nlm.nih.gov/#query=C10H16N2O2) | 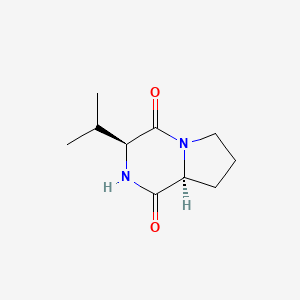 | potential antimicrobial agent of MRSA | https://link.springer.com/article/10.1007/s00203-020-01896-x |
| 12 | Phenol, 3,5-dimethoxy- / Phloroglucinol dimethyl ether / Taxicatigenin | 38 | 3.01 | [C_8_H_10_O_3_](https://pubchem.ncbi.nlm.nih.gov/#query=C8H10O3) | 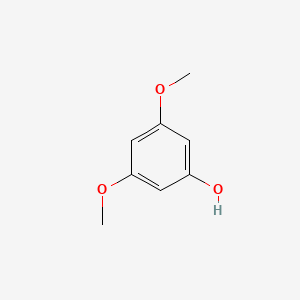 | Potential as antibacterial and antifungal plant material (*Taxus baccata*) | <https://sanitas.e-journal.id/index.php/SANITAS/article/view/20180601-irwandi/20180601-35-43>  <http://jddtonline.info/index.php/jddt/article/view/3635> |
| 13 | Pyrrolo[1,2-a]pyrazine-1,4-dione, hexahydro-3-(2-methylpropyl) | 91 | 3.27 | C_11_H_18_N_2_O_2_ | 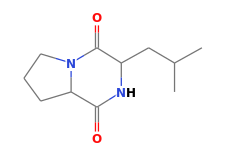 | Antioxidant activity | file:///C:/Users/A450L%20Series/Downloads/Marine_Sp_VITMK1_Derived_Pyrrolo_1_2-A_Pyrazine-1_.pdf |
| 14 | Pyrrolo[1,2-a]pyrazine-1,4-dione, hexahydro-3-(2-methylpropyl) | 95 | 4.26 | C_11_H_18_N_2_O_2_ | 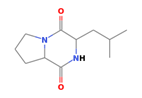 | Antioxidant activity | file:///C:/Users/A450L%20Series/Downloads/Marine_Sp_VITMK1_Derived_Pyrrolo_1_2-A_Pyrazine-1_.pdf |
| 15 | Pyrrolo[1,2-a]pyrazine-1,4-dione, hexahydro-3-(2-methylpropyl) | 93 | 3.63 | C_11_H_18_N_2_O_2_ | 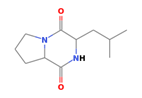 | Antioxidant activity | file:///C:/Users/A450L%20Series/Downloads/Marine_Sp_VITMK1_Derived_Pyrrolo_1_2-A_Pyrazine-1_.pdf |
| 16 | Pyrrolo[1,2-a]pyrazine-1,4-dione, hexahydro-3-(2-methylpropyl) | 80 | 2.27 | C_11_H_18_N_2_O_2_ | 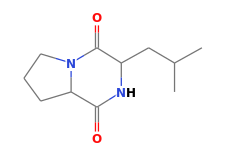 | Antioxidant activity | file:///C:/Users/A450L%20Series/Downloads/Marine_Sp_VITMK1_Derived_Pyrrolo_1_2-A_Pyrazine-1_.pdf |
| 17 | Butalbital / Barbituric acid, 5-a llyl-5-isobutyl / Alisobumal | 55 | 0.66 | C_11_H_16_N_2_O_3_ | 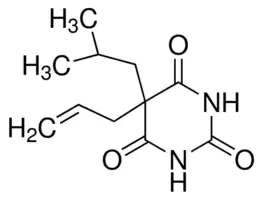 | used for treatment of pain and headache. It has a role as a sedative and an analgesic. | National Center for Biotechnology Information (2022). PubChem Compound Summary for CID 2481, Butalbital. Retrieved July 18, 2022 from https://pubchem.ncbi.nlm.nih.gov/compound/Butalbital. |
| 18 | 2-HYDROXY-3,5,5-TRIMETHYL-2-CYCLOH EXENONE / 2-Hydroxyisophorone | 49 | 2.37 | [C_9_H_14_O_2_](https://pubchem.ncbi.nlm.nih.gov/#query=C9H14O2) | 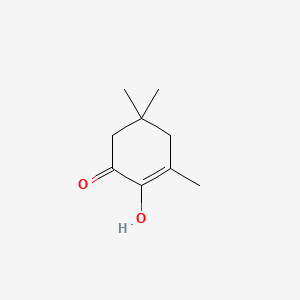 | Antimicrobial activity | https://link.springer.com/article/10.1007/s00284-020-02335-2#citeas |
| 19 | 2-HYDROXY-3,5,5-TRIMETHYL-2-CYCLOH EXENONE / 2-Hydroxyisophorone | 60 | 0.65 | [C_9_H_14_O_2_](https://pubchem.ncbi.nlm.nih.gov/#query=C9H14O2) | 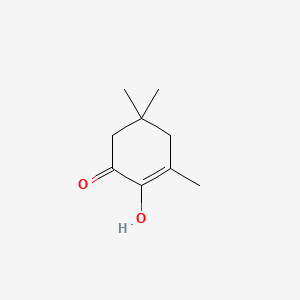 | Antimicrobial activity | https://link.springer.com/article/10.1007/s00284-020-02335-2#citeas |
| 20 | 4-(Phenylvinyl)nicotinaldehyde | 64 | 8.41 | C_6_H_5_NO | 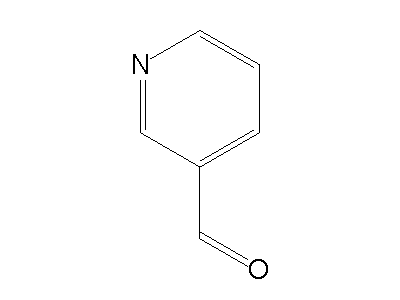 | - | - |
| 21 | Pyrrolo[1,2-a]pyrazine-1,4-dione, hexahydro-3-(phenylmethyl) | 91 | 3.38 | [C_14_H_16_N_2_O_2_](https://pubchem.ncbi.nlm.nih.gov/#query=C14H16N2O2) | 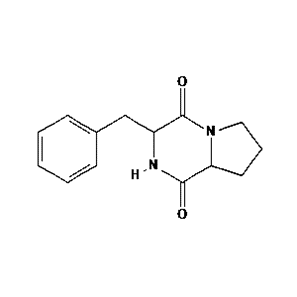 | Antioxidant activity | file:///C:/Users/A450L%20Series/Downloads/Marine_Sp_VITMK1_Derived_Pyrrolo_1_2-A_Pyrazine-1_.pdf |
| 22 | Pyrrolo[1,2-a]pyrazine-1,4-dione, hexahydro-3-(phenylmethyl) | 98 | 9.31 | [C_14_H_16_N_2_O_2_](https://pubchem.ncbi.nlm.nih.gov/#query=C14H16N2O2) | 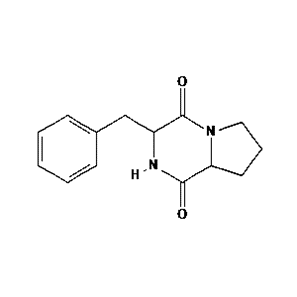 | Antioxidant activity | file:///C:/Users/A450L%20Series/Downloads/Marine_Sp_VITMK1_Derived_Pyrrolo_1_2-A_Pyrazine-1_.pdf |
| 23 | 2-Methyl-5H-dibenz[b,f]azepine | 50 | 0.96 | [C_15_H_13_N](https://pubchem.ncbi.nlm.nih.gov/#query=C15H13N) | 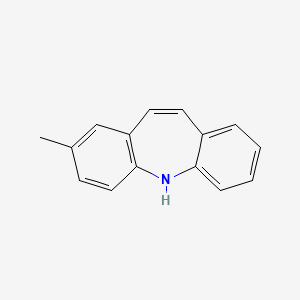 | - | - |
| 24 | 1,3-dimethyl-4-azaphenanthrene | 25 | 0.12 | - | - | - | - |
| 25 | Furazano[3,4-d]pyrimidine-5,7(4H,6H)-dione | 43 | 8.48 | - | - | - | - |
| 26 | 1,3-Cyclohexanedione, 2,5,5-trimethyl- (CAS) / 2-Methyldimedone | 52 | 10.82 | C_9_H_14_O_2_ | 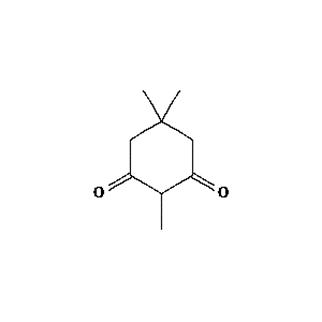 |  |  |
| 27 | 2-Methyl-5H-dibenz[b,f]azepine | 59 | 1.49 | [C_15_H_13_N](https://pubchem.ncbi.nlm.nih.gov/#query=C15H13N) | 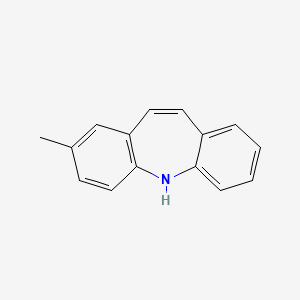 | - | - |
| 28 | 2-Methyl-5H-dibenz[b,f]azepine | 59 | 1.49 | [C_15_H_13_N](https://pubchem.ncbi.nlm.nih.gov/#query=C15H13N) | 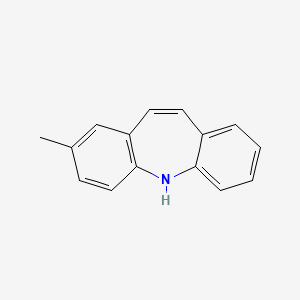 | - | - |
| 29 | 2-Methyl-5H-dibenz[b,f]azepine | 59 | 2.24 | [C_15_H_13_N](https://pubchem.ncbi.nlm.nih.gov/#query=C15H13N) | 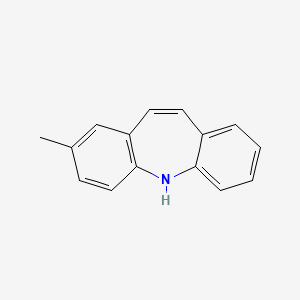 | - | - |
| 30 | 2-Methyl-5H-dibenz[b,f]azepine | 59 | 1.56 | [C_15_H_13_N](https://pubchem.ncbi.nlm.nih.gov/#query=C15H13N) | 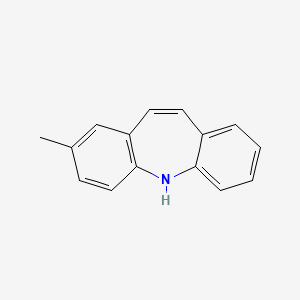 | - | - |
| 31 | 2-Methyl-5H-dibenz[b,f]azepine | 59 | 0.18 | [C_15_H_13_N](https://pubchem.ncbi.nlm.nih.gov/#query=C15H13N) | 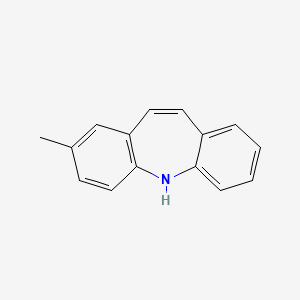 | - | - |

GC-MS profile of the active fraction from 1 AC crude extract

| **No** | **Name of the compound** | **Similarity Index** | **Area %** | **Molecular Formula** | **Structure** | **Biological Properties** | **References** |
| --- | --- | --- | --- | --- | --- | --- | --- |
| 1 | Piperidine, 2,6-dimethyl- (CAS) / 2,6-Dimethylpiperidine / Nanofin | 53 | 0.96 | [C_7_H_15_N](https://pubchem.ncbi.nlm.nih.gov/#query=C7H15N) | 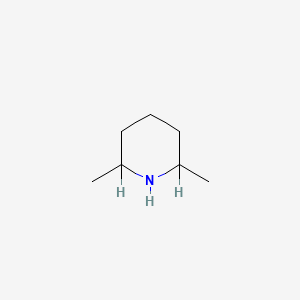 | antimicrobial,  anti-inflammatory, antiviral, antimalarial, general anesthetic,  antidepressant, antioxidant, antiepileptic, antitumor, anticonvulsant,  and antihyperlipidemic activitie | file:///C:/Users/A450L%20Series/Downloads/admin,+Journal+manager,+14_AJPCR_26536_REV%20(1).pdf |
| 2 | 4H-Pyran-4-one, 2,3-dihydro-3,5-dihydroxy-6-methyl- | 94 | 4.31 | [C_6_H_8_O_4_](https://pubchem.ncbi.nlm.nih.gov/#query=C6H8O4) | 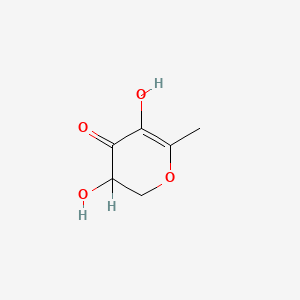 | Antioxidant activity | https://link.springer.com/article/10.1007/s00217-011-1527-4 |
| 3 | 3-Methyl-4-phenylpyrrole | 60 | 3.25 | [C_11_H_11_N](https://pubchem.ncbi.nlm.nih.gov/#query=C11H11N) | 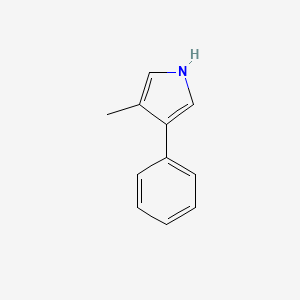 | 4-phenylpyrrole derivatives as novel androgen receptor antagonists | https://www.sciencedirect.com/science/article/abs/pii/S0968089611008947 |
| 4 | Coniine / (S)-2-Propylpiperidine / Piperidine, 2-propyl-, (S)- / Cicutin | 80 | 25.31 | [C_8_H_17_N](https://pubchem.ncbi.nlm.nih.gov/#query=C8H17N) | 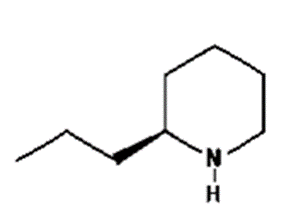 | treat herpes, erysipelas and St. Anthony's fire (a bacterial skin infection caused by *Streptococcus pyogenes*), breast tumors, inactive tumour, swelling, and joint pains. | doi: [10.3390/molecules22111962](https://doi.org/10.3390%2Fmolecules22111962) |
| 5 | trans-2-(.beta.-Diethylaminoethenyl)-.delta.(2)oxazoline | 38 | 1.87 | - | - | - | - |
| 6 | 2-Hydroxy-3,5,5-trimethyl-cyclohex-2-enone | 59 | 4.02 | [C_9_H_14_O_2_](https://pubchem.ncbi.nlm.nih.gov/#query=C9H14O2) | 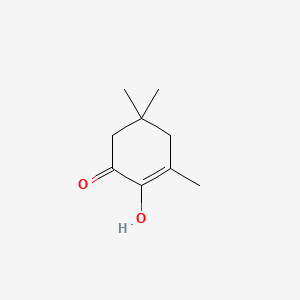 | Antimicrobial activity | https://link.springer.com/article/10.1007/s00284-020-02335-2#citeas |
| 7 | 2-Decene, 3-methyl-, (Z)- / (2Z)- 3-Methyl-2-decene | 72 | 3.20 | [C_11_H_22_](https://pubchem.ncbi.nlm.nih.gov/#query=C11H22) | 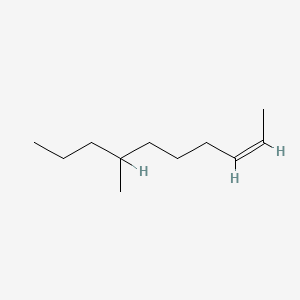 | - | - |
| 8 | Pyrrolo[1,2-a]pyrazine-1,4-dione, hexahydro-3-(2-methylpropyl) | 86 | 2.73 | C_11_H_18_N_2_O_2_ | 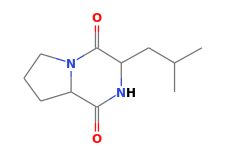 | Antioxidant activity | file:///C:/Users/A450L%20Series/Downloads/Marine_Sp_VITMK1_Derived_Pyrrolo_1_2-A_Pyrazine-1_.pdf |
| 9 | Pyrrolo[1,2-a]pyrazine-1,4-dione, hexahydro-3-(2-methylpropyl) | 95 | 3.47 | C_11_H_18_N_2_O_2_ | 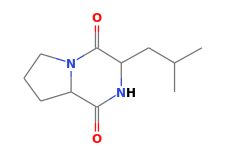 | Antioxidant activity | file:///C:/Users/A450L%20Series/Downloads/Marine_Sp_VITMK1_Derived_Pyrrolo_1_2-A_Pyrazine-1_.pdf |
| 10 | 3,9-DIAZATRICYCLO[7.3.0.0(3,7)]DOD ECAN-2,8-DIONE / Cyclo-L-prolyl-L-proline | 46 | 7.65 | [C_10_H_14_N_2_O_4_](https://pubchem.ncbi.nlm.nih.gov/#query=C10H14N2O4) | 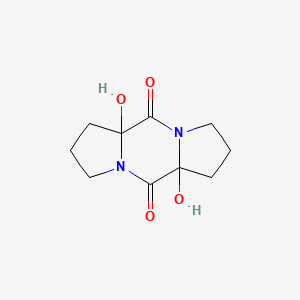 | - | - |
| 11 | trans-2-(.beta.-Diethylaminoethenyl) .delta.(2)oxazoline | 49 | 1.09 | - | - | - | - |
| 12 | 4-Methyl-2,7-dioxa-tricyclo[4.4.0.0 (3,8)]decane | 53 | 3.08 | [C_9_H_14_O_2_](https://pubchem.ncbi.nlm.nih.gov/#query=C9H14O2) | 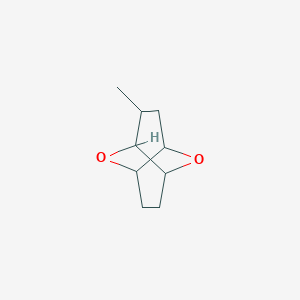 | - | - |
| 13 | 2-(3'-Hydroxypropyl)-3,5,6-trimethyl-1,4-benzoquinone | 83 | 15.47 | C_13_H_18_O_4_ | 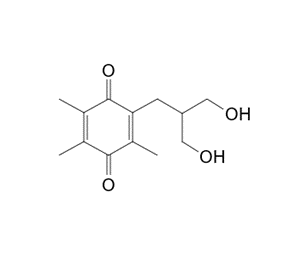 | - | - |
| 14 | Ergotamine | 81 | 3.38 | [C_33_H_35_N_5_O_5_](https://pubchem.ncbi.nlm.nih.gov/#query=C33H35N5O5) | 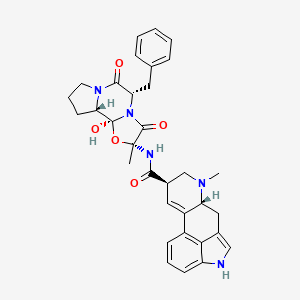 | Acute migraine drug (vasoconstrictor) | https://link.springer.com/referenceworkentry/10.1007/978-3-319-19456-1_1-1?noAccess=true |
| 15 | Pyrrolo[1,2-a]pyrazine-1,4-dione, hexahydro-3-(phenylmethyl) | 98 | 5.06 | [C_14_H_16_N_2_O_2_](https://pubchem.ncbi.nlm.nih.gov/#query=C14H16N2O2) | 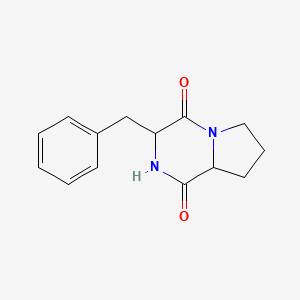 | Antioxidant activity | file:///C:/Users/A450L%20Series/Downloads/Marine_Sp_VITMK1_Derived_Pyrrolo_1_2-A_Pyrazine-1_.pdf |
| 16 | 4-Methyl-2,7-dioxa-tricyclo[4.4.0.0 (3,8)]decane | 50 | 3.76 | [C_9_H_14_O_2_](https://pubchem.ncbi.nlm.nih.gov/#query=C9H14O2) | 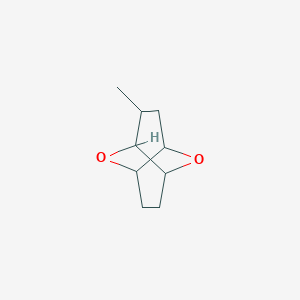 | - | - |
| 17 | 8-Dodecen-1-ol, (Z)- / Z-8-Dodecen-1-ol / (8Z)-8-Dodecen-1-ol | 55 | 3.87 | [C_12_H_24_O](https://pubchem.ncbi.nlm.nih.gov/#query=C12H24O) | 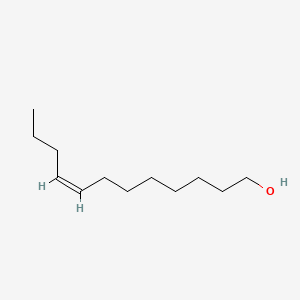 | - | - |
| 18 | Phenol, 2,6-dimethoxy- (CAS) / 2,6-Dimethoxyphenol / DIMETHOXY PHENOL | 45 | 6.99 | [C_8_H_10_O_3_](https://pubchem.ncbi.nlm.nih.gov/#query=C8H10O3) | 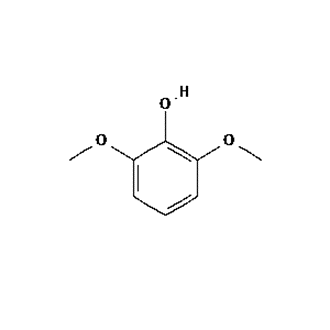 | Potential as antibacterial and antifungal plant material (*Taxus baccata*) | <https://sanitas.e-journal.id/index.php/SANITAS/article/view/20180601-irwandi/20180601-35-43>  <http://jddtonline.info/index.php/jddt/article/view/3635> |
| 19 | 2-Methyl-5H-dibenz[b,f]azepine | 59 | 0.52 | [C_15_H_13_N](https://pubchem.ncbi.nlm.nih.gov/#query=C15H13N) | 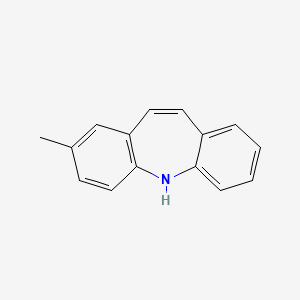 | - | - |
